# A Transcriptome Sequencing Study on Genome-Wide Gene Expression Differences of Lung Cancer Cells Modulated by Fucoidan

**DOI:** 10.3389/fbioe.2022.844924

**Published:** 2022-03-01

**Authors:** Yanjie Zhao, Xinmei Li, Heng Zhang, Mingzhe Yan, Mengmeng Jia, Qihui Zhou

**Affiliations:** ^1^ Institute for Translational Medicine, The Affiliated Hospital of Qingdao University, Qingdao University, Qingdao, China; ^2^ School of Public Health, Qingdao University, Qingdao, China

**Keywords:** marine biopolysaccharide, fucoidan, cancer, lung tumor, transcriptome sequencing

## Abstract

Fucoidan has received increasing attention in anti-(lung) tumors. However, the effect of fucoidan on the gene changes of lung cancer cells (LCCs) has not been examined systematically. Herein, we investigate the effect of fucoidan on the phenotypes of LCCs and their gene expression by transcriptome sequencing analysis. The phenotypes of LCCs are significantly inhibited by fucoidan. Importantly, compared to LCCs, 1 mg/ml fucoidan has no effect on the phenotypes of normal cells. Further, 6,930 differentially expressed genes (DEGs) in the transcriptome of LCCs (3,501 up-regulated and 3,429 down-regulated genes) are detected *via* RNA-sequencing between the fucoidan and control groups. Gene Ontology analysis confirms that DEGs are reflected in DNA replication, cell-substrate junction, regulation of cell cycle phase transition, apoptosis, focal adhesion, cadherin binding, and cell adhesion molecule binding. Thus, our findings on the transcriptomic level highlight the therapeutic potential of fucoidan for lung cancer treatment.

## Introduction

In the last few decades, marine polysaccharides from algae have gained considerable attention from the fundamental research and practical biomedical applications owing to their inherent (bio)physicochemical characteristics, such as favorable biocompatibility and biodegradability, remarkable bioactivity, as well as outstanding structural functionalities (Bilal and Iqbal, 2020; Zheng et al., 2020; He et al., 2021; Jing et al., 2021; Yang et al., 2021). In particular, fucoidan is a water-soluble fucose-based sulfated polysaccharide, which has remarkable multiple bioactivities, including antioxidant, antimicrobial, antithrombotic, anticoagulant, anti-inflammatory, antifibrotic, immunomodulatory, and antitumor functions (Lin et al., 2020a; Apostolova et al., 2020; Etman et al., 2020; Hao et al., 2020; Zhu et al., 2021a; Hao et al., 2021; Zheng et al., 2021). Among them, its characteristic of anti-cancer cells makes it a promising candidate in the therapy of tumors, which has recently attracted increasing attention in healthy food and biomedicine (Chung et al., 2020; Etman et al., 2020; Oliveira et al., 2020). The antitumor feature of fucoidan has been reported for various cancers *in vitro* and *in vivo* (Lin et al., 2020b).

Among them, lung cancer is the second most diagnosed tumor and one of the deadliest cancers around the world, resulting in long-standing cough, chest infections, persistent and breathlessness (Minna et al., 2002; Herbst et al., 2018; Howlader et al., 2020). Conventional chemotherapy has serious side effects, such as lowered white blood cell counts, increased risk of infection, and issues with heart function (Chen et al., 2006; Lee et al., 2014). Therefore, it is vital to find naturally derived anti-cancer agents with no or minimal side effects. As reported, fucoidan has the ability to effectively destroy lung cancer cells without significant side effects (Oliveira et al., 2020; Qiu et al., 2020). For instance, Lee *et al.* reported that fucoidan had the anti-metastatic ability on lung cancer cells (A549 cell line) through affecting ERK1/2, Akt-mTOR, and NF-κB signaling pathways, inhibiting the migration and invasion of A549 cells (Lee et al., 2012). Boo et al. found that fucoidan caused apoptosis of A549 cells *via* hindering p38 and PI3K/Akt as well as inducing the ERK1/2 MAPK pathway (Boo et al., 2011). However, the effect of fucoidan on the gene changes of lung cancer cells has not been examined systematically in a high-throughput manner. Elucidating how fucoidan modulates the responses of lung cancer cells plays a critical role in treating lung tumors efficiently.

Inspired by the observations above, in this work, fucoidan was used to investigate its effect on the phenotypes of lung cancer cells and their gene expression by transcriptome sequencing analysis. The effects of fucoidan on the adhesion, morphology, proliferation, and migration of normal and lung cancer cells were detected. Particularly, the transcriptomics analysis of lung cancer cells was performed.

## Materials and Methods

### Materials

Fucoidan derived from Undaria pinnatifida [Molecular weight (Mw) = 276 kDa, purity ≥95%, sulfate: 29.65% (Table 1)] was provided by Qingdao Bright Moon Seaweed Group Co., Ltd. (China). Dextran sulfate and monosaccharide standards, including rhamnose, galactose, glucose, arabinose, xylose, mannose, and fucose, were supplied by Sigma Chemical Co., Ltd. (United States). Human lung squamous cell line (H226) and bronchial epithelial cells (16HBE) were obtained from the American Type Culture Collection (ATCC, Manassas, United States). All chemicals were used as received without further purification.

**TABLE 1:** The Mw and chemical composition of fucoidan.

Samples	Mw (kDa)	Total sugar	Sulfate (%)	Uronic acid (%)	Monosaccharide^a^ (%)				
					L-fucose	Galactose	Mannose	Rhamnose	D-glucose
Fucoidan	276	78.61	29.65	2.01	54.26	42.19	1.6	1.1	0.85

a Taking total monosaccharide as 100%.

### Cell Culture

16HBE, a commonly used lung bronchial epithelial cell, was represented as normal lung cell in this study. H226, a commonly used lung squamous cell carcinoma cell, was represented cancer cells in this study. These cells were incubated in MEM/EBSS or RPMI-1640 Medium (Hyclone) containing 10% fetal bovine serum (FBS, Gibco, Amarillo, TX), 0.1 mg/ml streptomycin (ThermoFisher, United States), and 100 U/mL penicillin (ThermoFisher, United States) at 37 °C in a humidified 5% CO_2_ atmosphere. Fucoidan was dissolved in a serum-free medium and then sterilized by a 0.45 µm filter. The cells were co-cultured with the above fucoidan solution with various concentrations (i.e., 0, 1, 10, and 100 mg/ml).

### Cell Adhesion Assay

Cells were seeded in 96-well plates with a density of 2 × 10^4^ cells/well and incubated with fucoidan/medium solutions for 1 day. Cells were fixated with 4% paraformaldehyde for 10 min and then rinsed three times with PBS. Afterward, cells were permeabilized with Triton X-100 (Solarbio, Beijing, China) at 0.5% (v/v) for 5 min, and then rinsed three times with PBS. Finally, the cells were stained with FITC-Phalloidin and DAPI (Solarbio, Beijing, China) and imaged by the High Content Analysis System-Operetta CLS™ (PerkinElmer, Waltham Mass, United States). The expression of the actin cytoskeleton, cell density and elongation were analyzed by ImageJ software. Firstly, open the figure in the software and set it to 8-bit format. Then select the parameters such as area, integrated density to be measured, and analyze to obtain quantitative data.

### Cell Proliferation Assay

Cells were seeded in 96-well plates with a density of 3,000 cells/well and incubated with fucoidan/medium solutions for 1, 3, and 5 days. Afterward, the medium was exchanged with a fresh medium containing cell counting kit 8 (Dojindo labs, Kumamoto, Japan). The ratio of serum-free medium to CCK8 was 10:1 and then incubated for 2 h at 37°C. The optical density (OD) values were measured using a microplate reader (SynergyH1/H1M, Bio-Tek, China) at 450 nm.

### Cell Migration Assay

H226 cells were seeded in 12-well plates with 2.5 × 10^4^ cells/well and cultured for 1 day to reach confluence. Afterward, a 200 µL pipette tip was used to make a straight scratch. Images at 0, 12, 24, and 48 h after scratches were collected using the Olympus inverted fluorescence microscope (Olympus, Tokyo, Japan), and the results were calculated with ImageJ software.

### Transcriptome Analysis

Illumina transcriptome sequencing (Novogene, Beijing, China) was performed on abundant H226 cells co-cultured with different concentrations (0 and 1 mg/ml) of fucoidan. Next, differentially expressed genes (DEGs) were analyzed using DESeq2 1.16.1 software. We used |log2(Fold Change) |>0 & *p* < 0.05 as the screening criteria. Results of DEGs were presented as a volcano plot and a heatmap. Moreover, to analyze the DEGs at the functional level, Genetic Ontology (GO) and Kyoto Encyclopedia of Genes and Genomics (KEGG) analyses were performed using the DAVID database as previously described (Ju et al., 2019). There were three biological replicates in each group.

### Statistical Analysis

All data were shown as mean ± standard deviation (SD). Statistical analyses were evaluated using Graphpad Prism 8. The student’s *t-tests* were used to determine the difference between the two groups. A value of *p* < 0.05 was considered to be statistically significant.

## Results and Discussion

### Effect of Fucoidan on the Adhesion of H226 and 16HBE Cells

**FIGURE 1:**
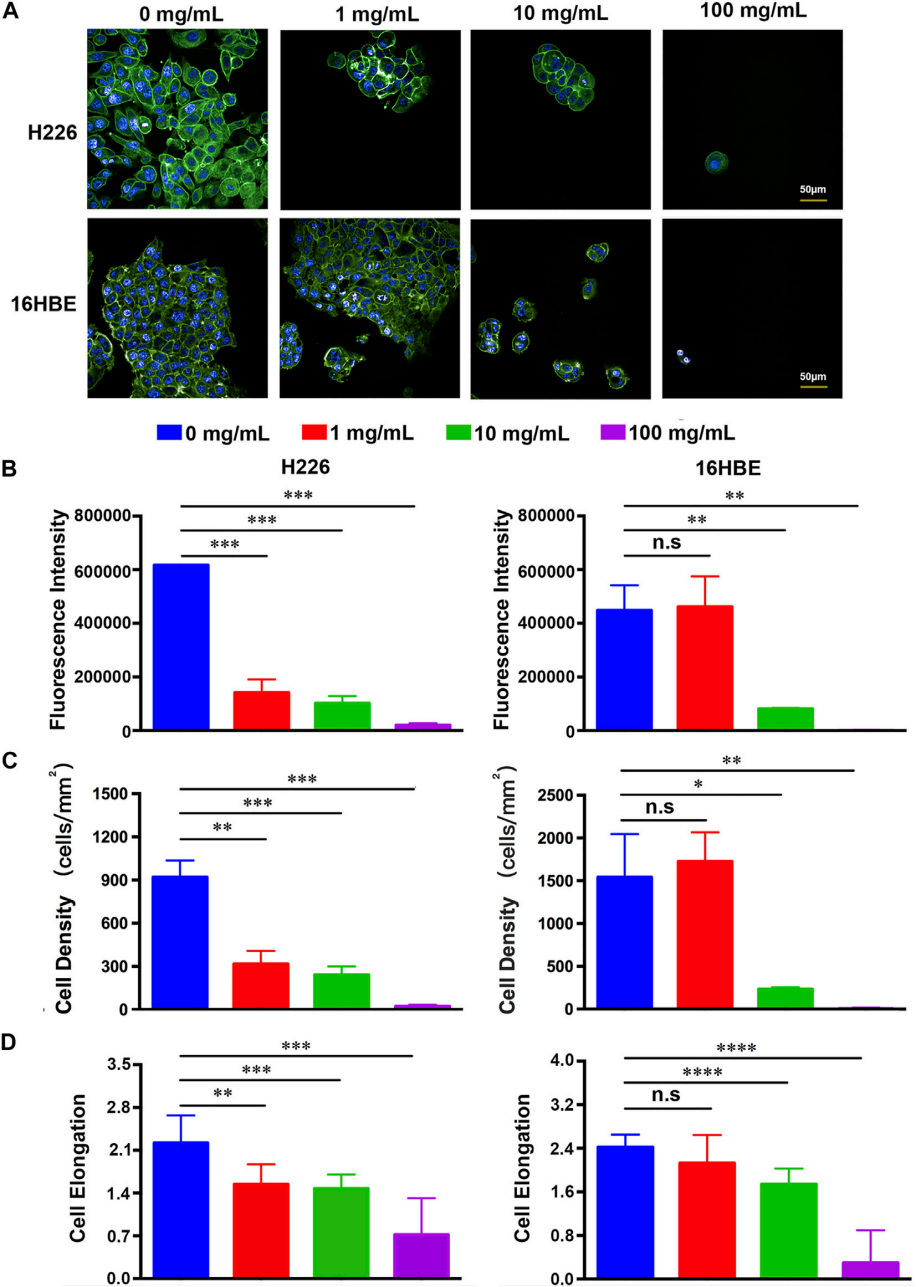
**(A)** Fluorescence images of H226 and 16HBE cells treated with different concentrations of fucoidan. **(B–D)** Quantitative results of the expression of the actin cytoskeleton, cell density and elongation in H226 and 16HBE.

Cellular adhesion has been considered as the initial and critical response of the cell with its surrounding microenvironment, which determines the subsequent behaviors of the cell, such as morphology change, migration, proliferation, and functionalization (Zhou et al., 2018; Li et al., 2019; Mierke, 2020; Zhou et al., 2020). The adhesion of H226 and 16HBE cells in different concentrations of fucoidan solutions after 1day of cell culture was investigated with a double-label fluorescence staining of the actin cytoskeleton (green) and nucleus (blue). As shown in Figure 1A, compared to the control group, the number of attached H226 cells significantly reduced and cell spreading also decreased in 1 mg/ml fucoidan, while the morphology and adhesion of 16HBE cells were not affected in 1 mg/ml fucoidan. It indicates that the effect of 1 mg/ml fucoidan on H226 cells may specifically be modulated by attenuating the attachment of cancer cells but not by cytotoxicity. With increasing the fucoidan concentration from 1 to 100 mg/ml, for both types of cells, the expression of the actin cytoskeleton, cell density and elongation greatly decreased.

To better understand the effect of fucoidan on cell adhesion, the expression of the actin cytoskeleton, cell density and elongation were quantified by the analysis of the positively stained cells using ImageJ software as indicated in Figures 1B–D. For H226 cells, the expression of the actin cytoskeleton, cell density and elongation greatly decreased with an increased concentration of fucoidan, suggesting that fucoidan remarkably inhibited cancer cell adhesion. For 16HBE cells, the expression of the actin cytoskeleton, cell density and elongation in 1 mg/ml fucoidan had no significant difference with the blank control. However, with increasing the fucoidan concentration from 1 to 100 mg/ml, the cell parameters greatly reduced, which reveals that high fucoidan concentration suppressed 16HBE cells adhesion. This phenomenon may be due to the down-regulation of adhesion signal by 1 mg/ml fucoidan in cancer cells. These results are consistent with previous reports. Zhang reported that fucoidan inhibited osteosarcoma cell adhesion by suppression of the phosphorylation of FAK and paxillin (Zhang et al., 2020). In addition, fucoidan suppressed mouse hepatocarcinoma Hca-F cells adhesion *via* downregulating L-selectin and upregulating protein levels of tissue inhibitor of metalloproteinases (TIMPs) (Wang et al., 2014). Taken together, these results indicate that 1 mg/ml fucoidan could significantly suppress cancer cell adhesion but had no effect on normal cells.

### Effect of Fucoidan on the Proliferation of H226 and 16HBE Cells

**FIGURE 2:**
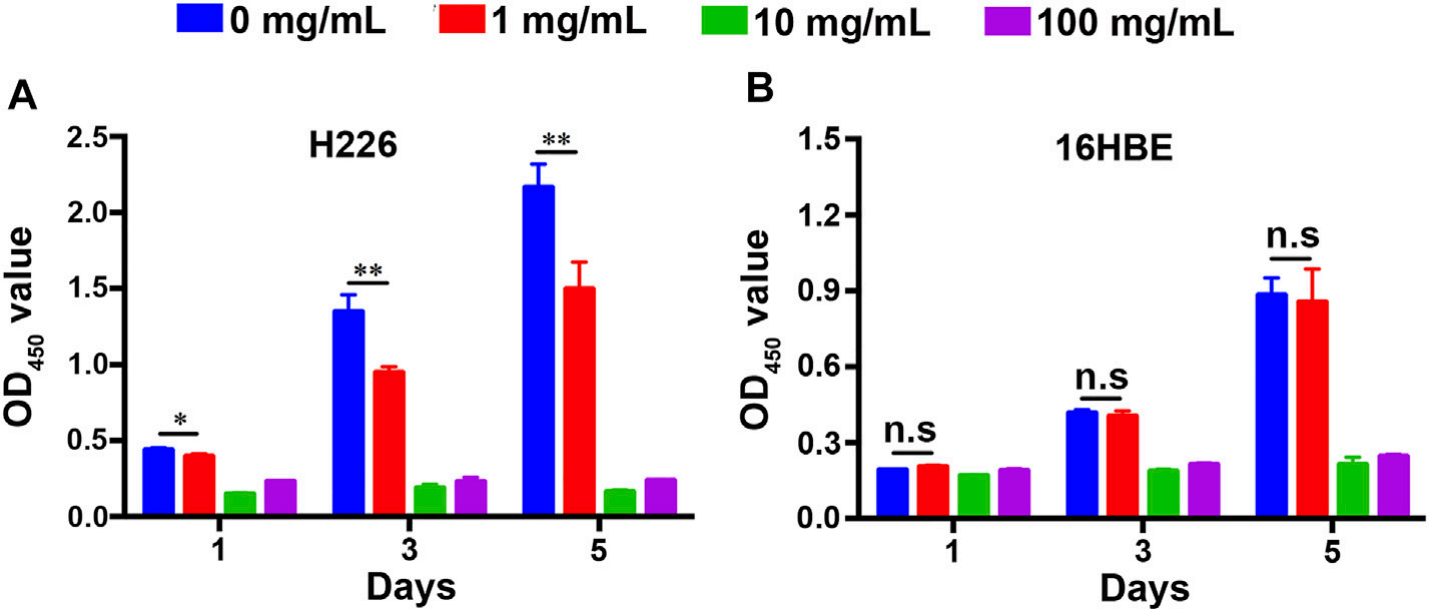
Cell proliferation of **(A)** 16HBE and **(B)** H226 cells after being treated with different concentrations of fucoidan in 1, 3, and 5 days.

Unregulated proliferation is considered a key hallmark of cancer development and progression (Hanahan and Weinberg, 2011). Inhibiting cancer cell proliferation is fundamental to cancer therapy (Chen et al., 2021). To detect the effect of fucoidan on the proliferation of H226 and 16HBE cells, a CCK-8 viability assay was performed on 1, 3, and 5 days as shown in Figure 2. It was found that the viability of H226 greatly reduced with the increment of fucoidan concentration, indicating that fucoidan could significantly inhibit the proliferation of cancer cells (Figure 2A). Particularly, when the fucoidan concentration increased from 1 to 10 mg/ml, the viability of H226 decreased abruptly. However, there was no significant difference between 10 and 100 mg/ml.

Although inhibition of cancer cell proliferation is crucial, we expect it would not affect the proliferation and activity of normal cells. We tested the effect of the fucoidan on the proliferation of 16HBE. Interestingly, compared to the blank control, 1 mg/ml fucoidan had no effect on the viability of 16HBE. When the fucoidan concentration increased from 1 to 10 mg/ml, the viability of 16HBE reduced abruptly. However, there was no significant difference between 10 mg/ml and 100 mg/ml, which is a similar trend to the result of H226 (Figure 2B). These may be due to the effect of 10 mg/ml and 100 mg/ml fucoidan on cells is caused by its own toxicity. Taken together, 1 mg/ml fucoidan could be a suitable concentration that significantly inhibited the proliferation of H226, and had no effect on the proliferation of 16HBE. These results suggest that 1 mg/ml fucoidan may specifically interact with the proliferation-related genes in H226 cells, but not cytotoxicity.

### Effects of Fucoidan on the Migration of H226 Cells

**FIGURE 3:**
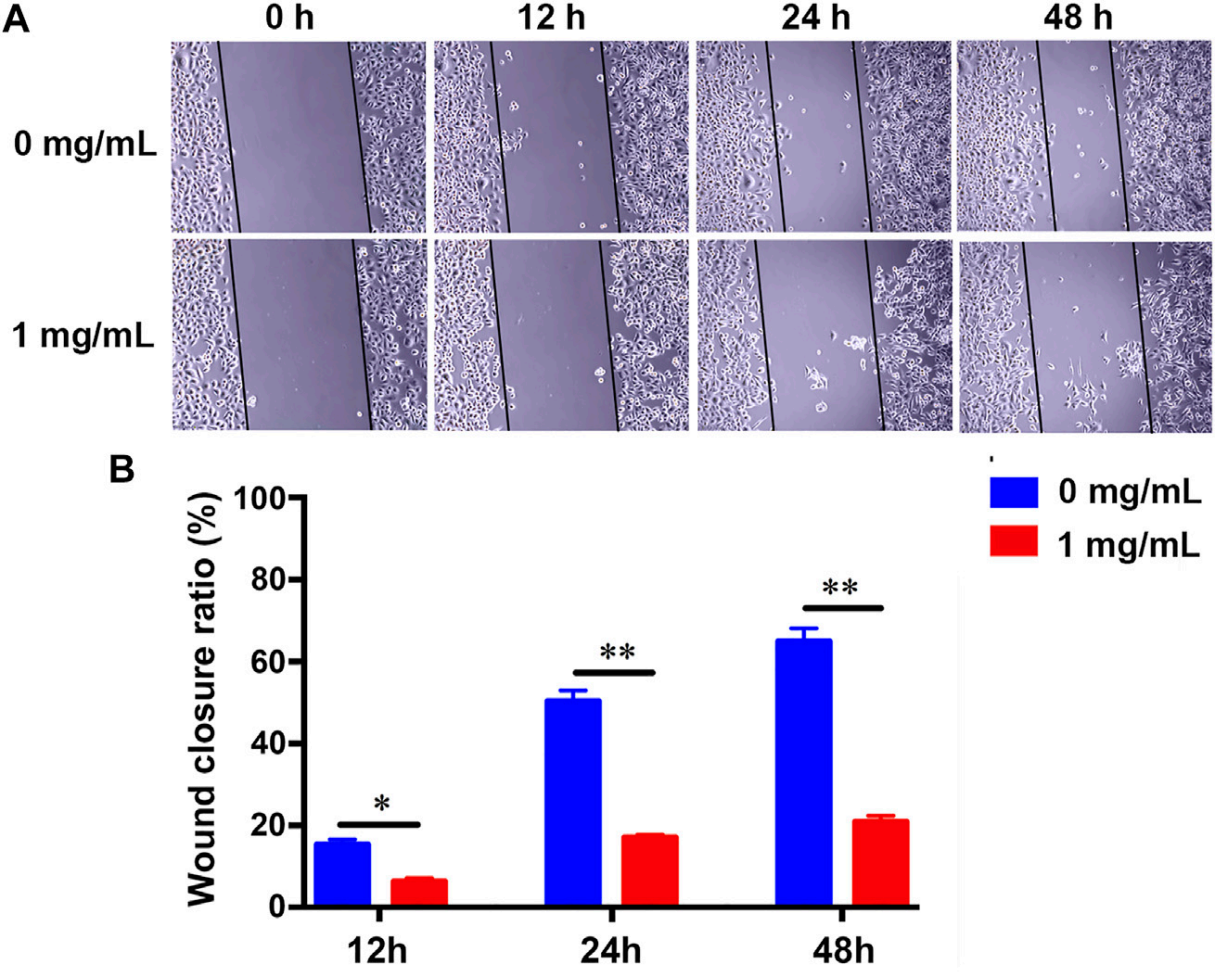
**(A)** The migration changes of H226 with the treatment of 1 mg/ml fucoidan by wound-healing assay. **(B)** Wound closure ratio of H226 after treated with 1 mg/ml fucoidan in 12, 24, and 48 h.

The invasion and metastasis of cancer cells is another major feature of cancer development and progression (Hanahan and Weinberg, 2011). Previous studies have reported that fucoidan could suppress the migration of cancer cells, such as gastric cancer (Xu et al., 2020), pancreatic cancer (Etman et al., 2021), and triple-negative breast cancer (Hsu et al., 2020). However, the effect of fucoidan in lung cancer is still unknown. A wound-healing assay was carried out to test the influence of 1 mg/ml fucoidan on the metastasis of lung cancer cells. It was found that the migration of H226 greatly reduced with the increment of the fucoidan concentration from 0 to 1 mg/ml (Figure 3A). Quantitative results showed 1 mg/ml fucoidan group significantly inhibited wound closure ratio compared with the blank control (Figure 3B). These results indicate that 1 mg/ml fucoidan could inhibit the migration of lung cancer cells.

### Effect of Fucoidan on the Gene Expression of H226 Cells

**FIGURE 4:**
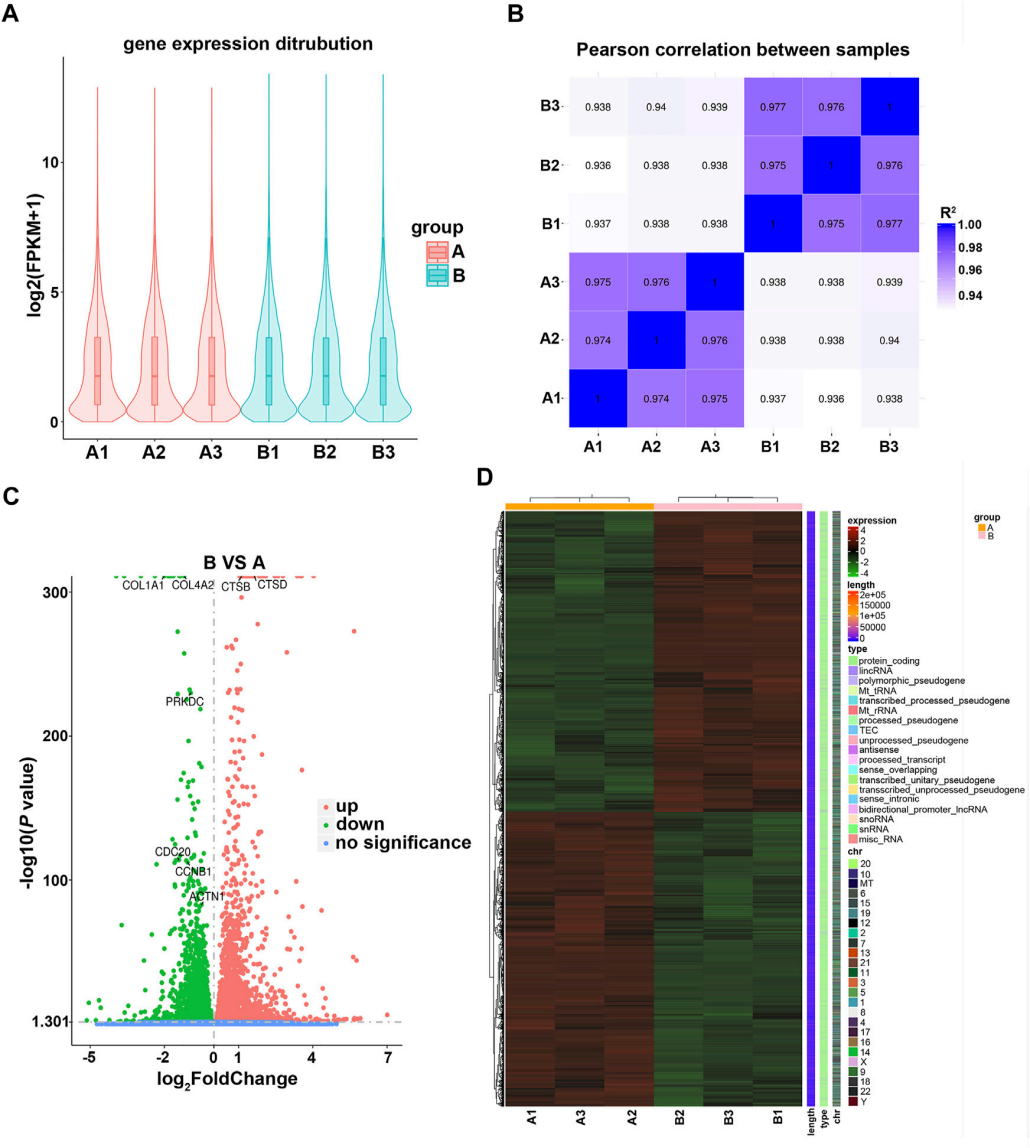
**(A)** Violin plot of the gene expression distribution between control and 1 mg/ml fucoidan exposed group. A1, A2, A3 indicate 0 mg/ml fucoidan exposed group; B1, B2, B3 indicate 1 mg/ml fucoidan exposed group. **(B)** The heat map of Pearson correlation between samples. **(C)** The volcano plots of DEGs. The red dots indicate upregulated genes, green dots indicate downregulated genes, and blue dots indicate non-differentially expressed genes. **(D)** The heat map of DEGs between the 0 mg/ml fucoidan exposed group and 1 mg/ml fucoidan exposed group.

To investigate the impact of fucoidan on genome-wide gene expression of lung cancer cells, RNA transcriptome sequencing was carried out on the H226 cells treated with 1 mg/ml fucoidan. To ensure the sequencing date reliable, we conducted quality control on the biological repetition of samples. The results show that there was no significant difference in the distribution of gene expression levels between the exposed 1 mg/ml fucoidan group and the blank control (Figure 4A). Moreover, analysis of inter-group sample difference and intra-group sample repetition showed that the correlation coefficient is close to 1 (Figure 4B). This indicates that the repeatability of the sample is good and the sequencing data is reliable.

Furthermore, DEGs were analyzed using DESeq2 software. Compared to the blank control, the volcano plot showed that 6,930 DEGs (3,501 up-regulated and 3,429 down-regulated genes) were identified (Figure 4C). Among these DEGs, some genes which with cell cycle (PRKDC, CDC20, CCNB1), cell apoptosis (CTSB, CTSD) and focal adhesion (COL4A2, COL1A1, ACTN1) were identified. Moreover, the DEGs were combined and analyzed by clustering. The heat map demonstrated that the DEGs were mainly protein-coding genes (Figure 4D). These results indicate that 1 mg/ml fucoidan may play anti-tumor roles by significantly modulating some protein-coding genes.

### Pathway Analysis of Fucoidan-Induced DEGs

**FIGURE 5:**
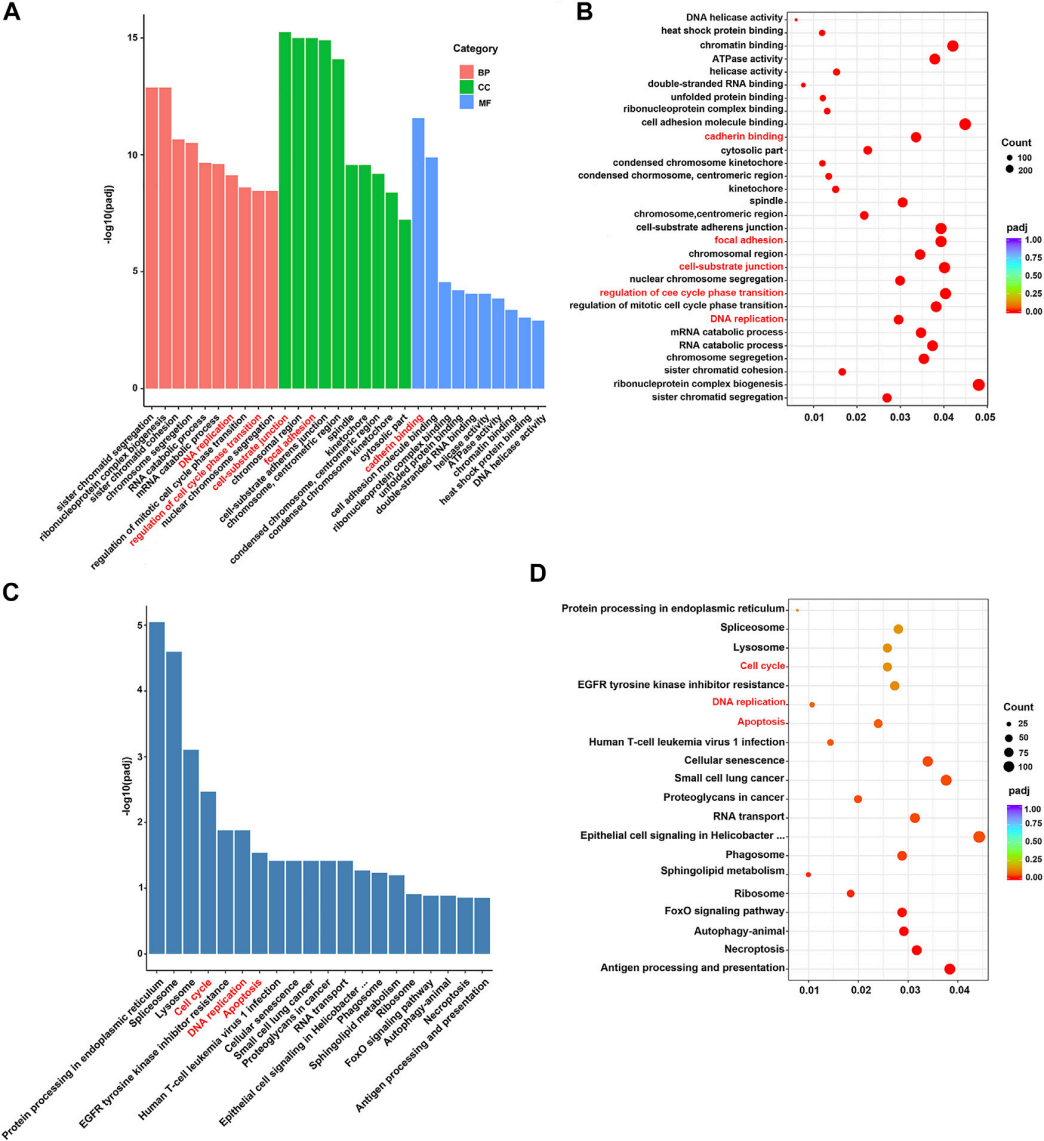
**(A,B)** GO functional enrichment analysis of DEGs. **(C,D)** KEGG pathway analysis of DEGs.

**FIGURE 6:**
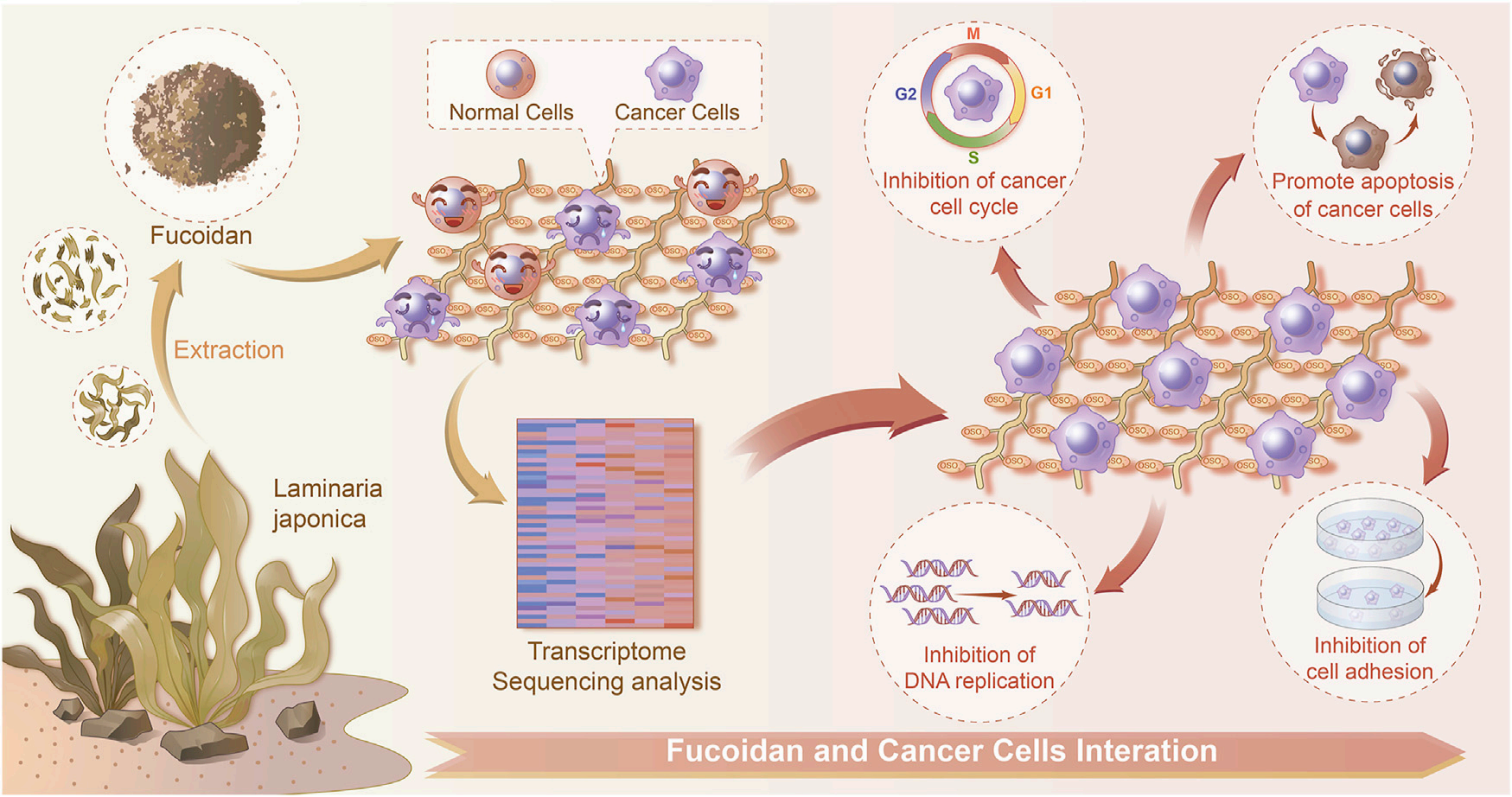
Schematic diagram of the interactions between normal/cancer cell and fucoidan detected by a transcriptome sequencing study.

To better understand the function of those DEGs regulated by fucoidan, GO and KEGG analysis was performed *via* DAVID. GO analysis indicated these DEGs were significantly enriched in many biological processes (BP), cellular component (CC), molecular function (MF), including DNA replication, cell-substrate junction, regulation of cell cycle phase transition, focal adhesion, cadherin binding, and cell adhesion molecule binding (Figures 5A,B). It has been well-demonstrated that DNA replication is one of the fundamental biological processes in which dysregulation can cause genome instability (Ubhi and Brown, 2019). DNA replication stress should drive cancer development and be considered a hallmark of cancer (Macheret and Halazonetis, 2015). Cell-substrate junction is associated with the EMT process and then affects cancer cells migration (Baronsky et al., 2017); (Paddillaya et al., 2019). Cell cycle phase transition is very finely regulated. The abnormal cell cycle, especially uncontrolled G1/S phase transition, is a key step in carcinogenesis (Rubin et al., 2020). Focal adhesion is a protein complex containing integrins that are regulated by a network of interactions between hundreds of proteins (Zhu et al., 2021b). The focal adhesion signal hub is composed of a variety of pro-survival signal molecules, including integrins and growth factor receptors, which strictly regulate cellular behavior, affect the survival of tumor cells (Eke and Cordes, 2015). Cadherin is a class of Ca^2+^ dependent transmembrane glycoproteins that mediate intercellular adhesion and play an important role in maintaining cellular polarity and maintaining the stability of intercellular adhesion (Nose and Takeichi, 1986). In tumor cells, the loss of this connective complex leads to the decrease of cell-cell adhesion, promotes the detachment of tumor cells from the primary lesion and the ability to cross the basal membrane, which is conducive to tumor metastasis (Kaszak et al., 2020). Adhesion-mediated cell adhesion is a key step in cancer invasion and metastasis, including integrins, selectin, cadherin, immunoglobulin superfamily, and CD44 (Mousa, 2010). Moreover, KEGG analysis showed the top 20 pathways, including “cell cycle”, “DNA replication”, and “apoptosis” (Figures 5C, D). Apoptosis is an active programmed cell death, which ensures a homeostatic balance between the rate of cell formation and cell death (Obeng, 2021). Once this balance is broken, it will lead to cancer. Recent studies showed apoptosis is the main way of cell death induced by various anticancer drugs (Ball and Borthakur, 2020; Tanaka et al., 2021). All these suggest fucoidan may induce dysregulation of DNA replication and cell cycle, reduce cell adhesion, cause cell death in the form of apoptosis, to achieve the effect of anti-cancer (Figure 6).

## Conclusion

In summary, the adhesion, proliferation, and migration of H226 cells as well as their gene expression were greatly modulated by fucoidan. Moreover, the H226 cell responses were significantly dependent on the concentration of fucoidan. Importantly, 1 mg/ml fucoidan suppressed lung cancer cell adhesion and proliferation but had no effect on normal cells. A transcriptome sequencing study demonstrated that DEGs were reflected in DNA replication, cell-substrate junction, regulation of cell cycle phase transition, focal adhesion, cadherin binding, and cell adhesion molecule binding. It was found that apoptosis and cell cycle-related DEGs were up-regulated in 1 mg/ml fucoidan. Thus, our work demonstrated on the transcriptomic level that fucoidan modulates the phenotype and gene expression of LCCs, displaying great potential for the treatment of lung tumors.

## Data Availability

The data presented in the study are deposited in the NCBI Sequence Read Archive (SRA) Database repository, accession number PRJNA801098.
