# Supplementary figures and images for: A Transcriptome Sequencing Study on Genome-Wide Gene Expression Differences of Lung Cancer Cells Modulated by Fucoidan
Source: Front Bioeng Biotechnol. 2022 Mar 1;10:844924. doi: 10.3389/fbioe.2022.844924 (PMC8923512; doi:10.3389/fbioe.2022.844924)

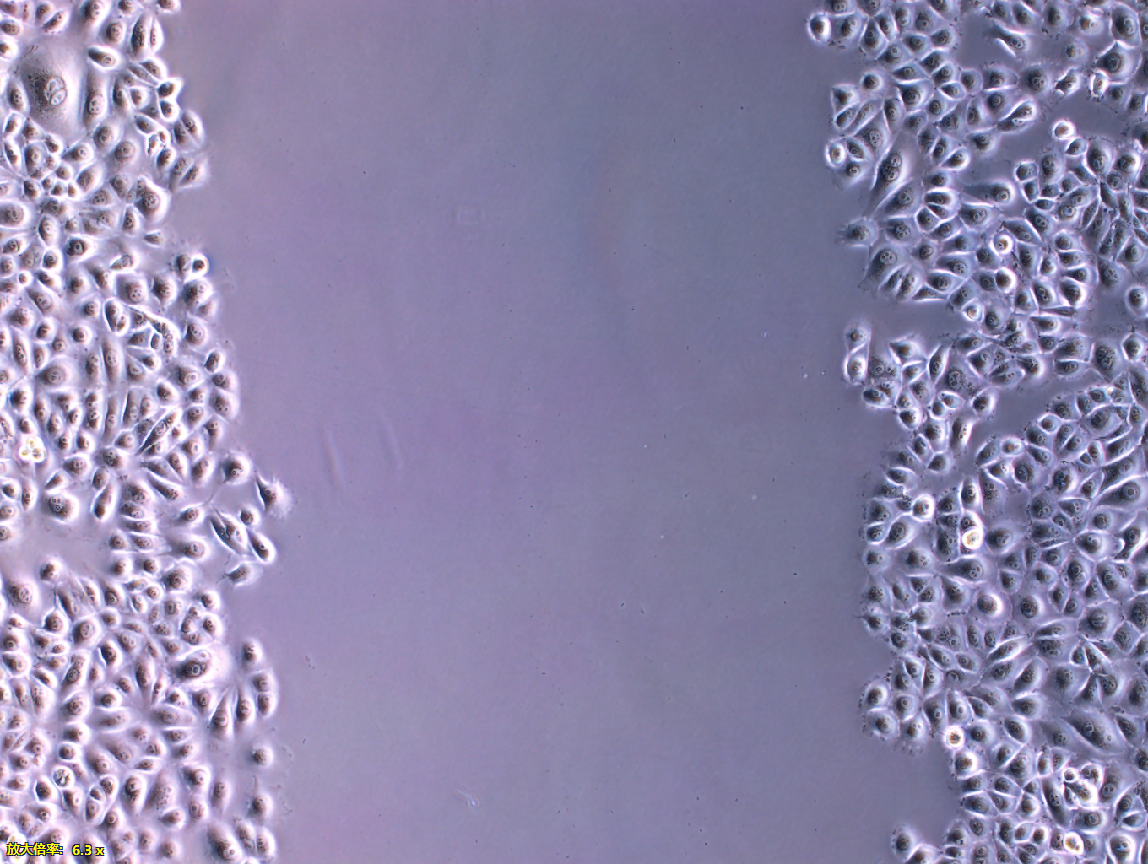

Supplement: Supplementary file 1 [file DataSheet3.ZIP › 0h/0-1.tif]

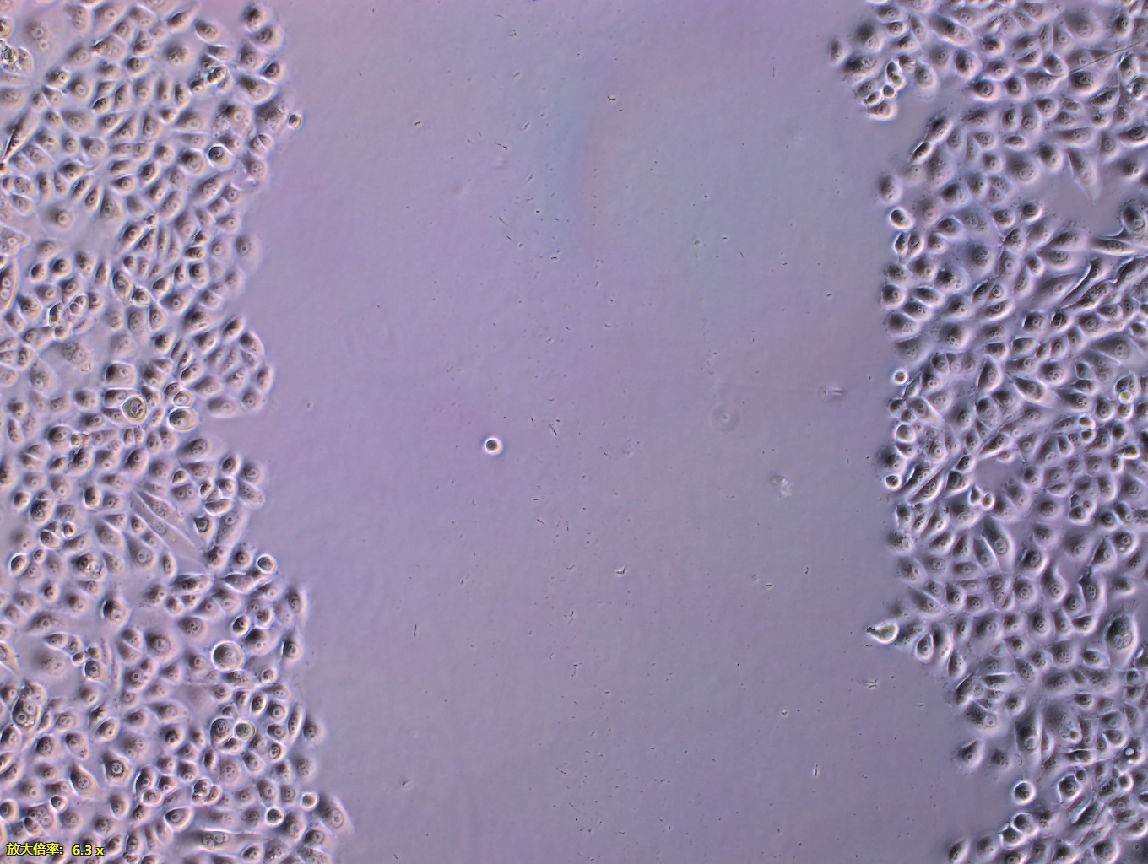

Supplement: Supplementary file 1 [file DataSheet3.ZIP › 0h/0-2.tif]

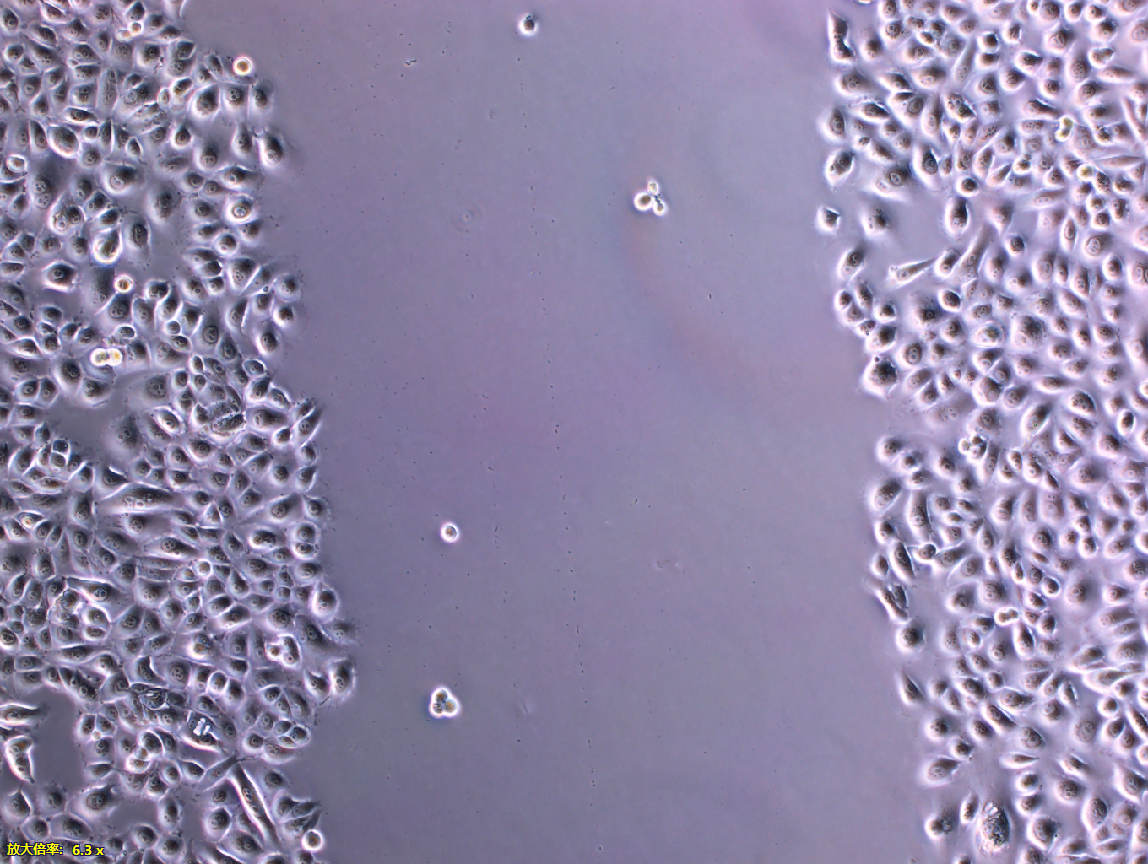

Supplement: Supplementary file 1 [file DataSheet3.ZIP › 0h/0.1-1.tif]

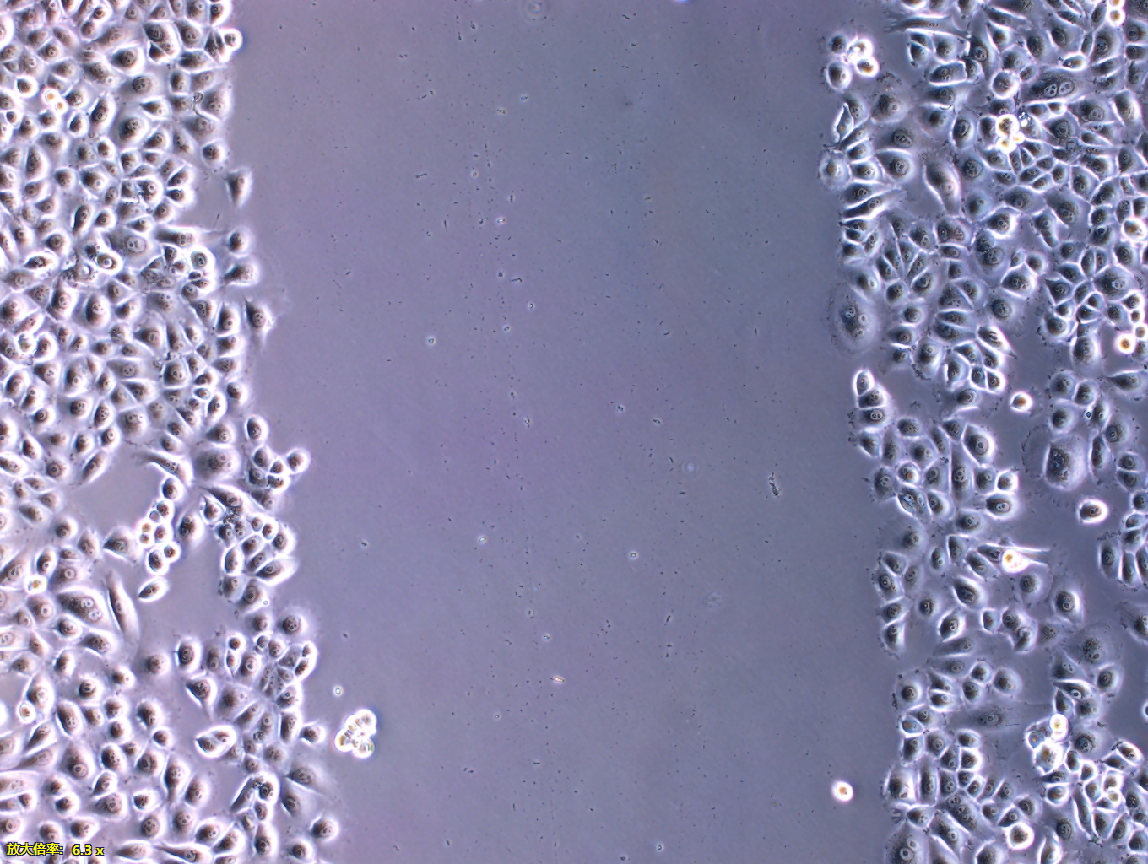

Supplement: Supplementary file 1 [file DataSheet3.ZIP › 0h/0.1-2.tif]

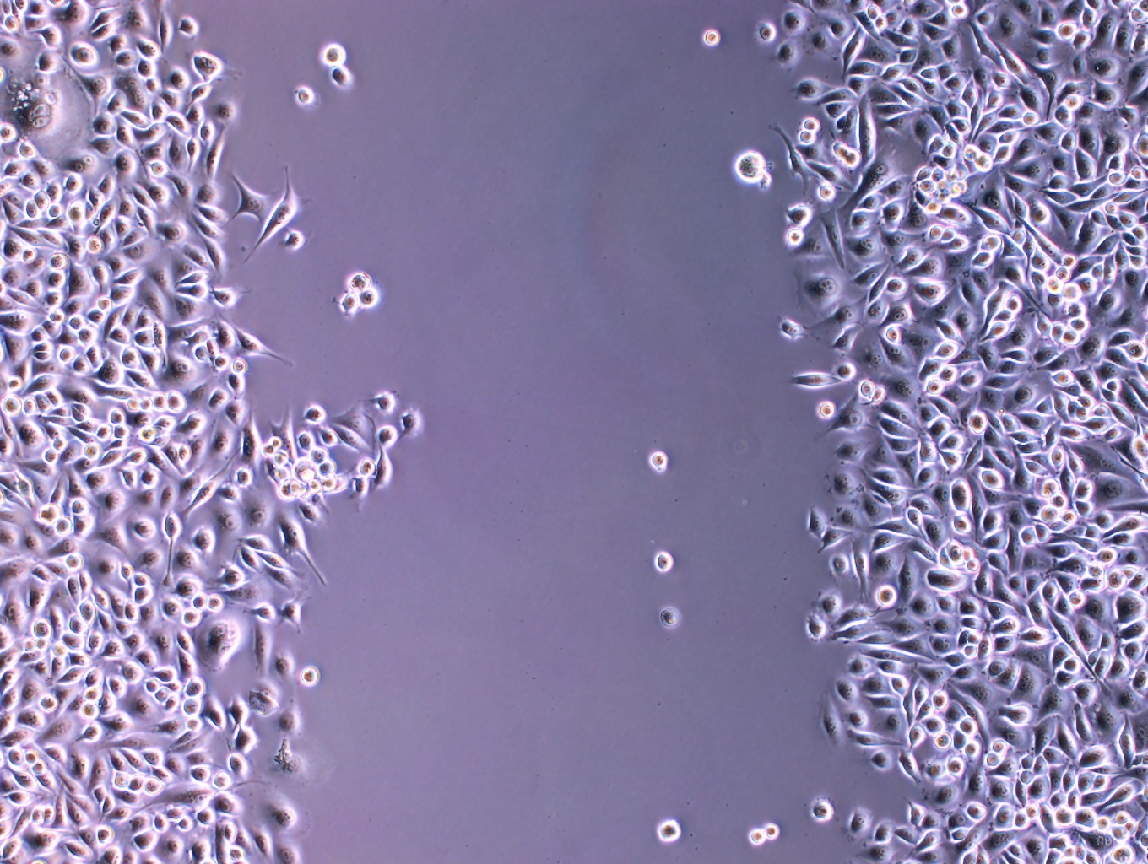

Supplement: Supplementary file 3 [file DataSheet4.ZIP › 12h/0-1.tif]

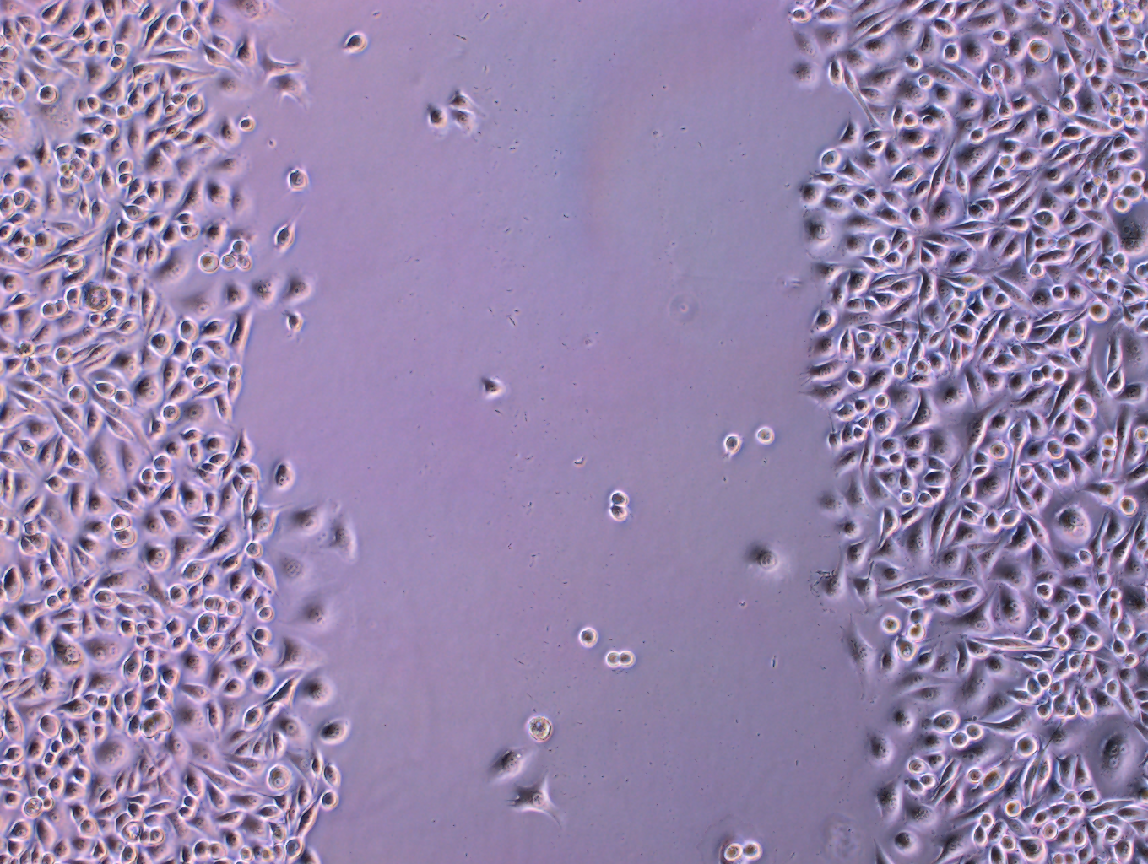

Supplement: Supplementary file 3 [file DataSheet4.ZIP › 12h/0-2.tif]

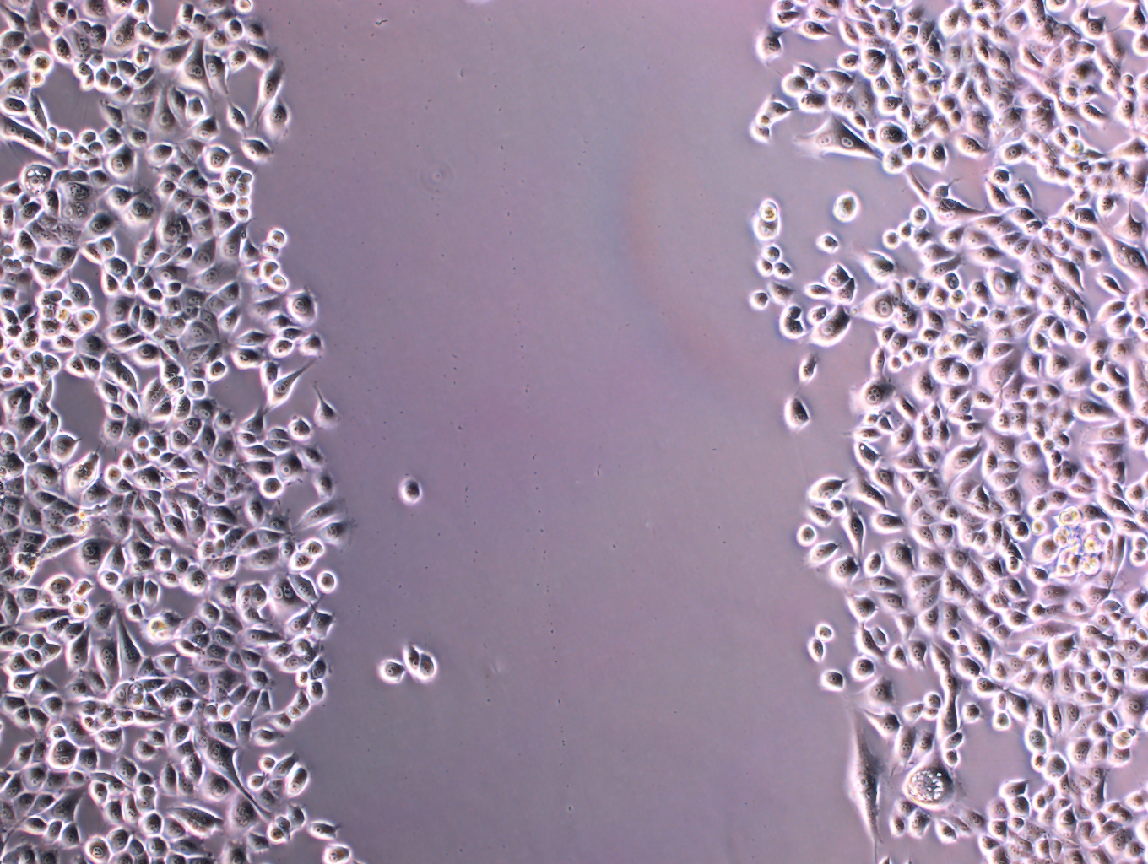

Supplement: Supplementary file 3 [file DataSheet4.ZIP › 12h/0.1-1.tif]

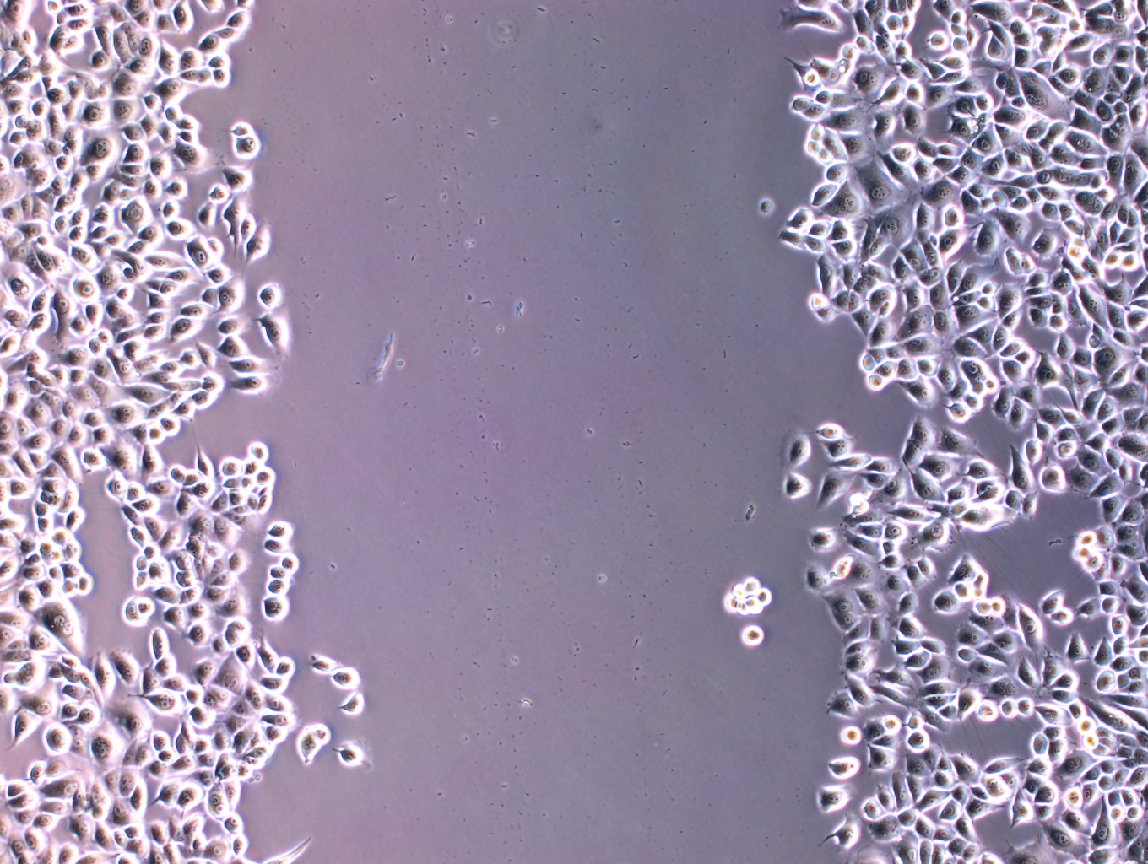

Supplement: Supplementary file 3 [file DataSheet4.ZIP › 12h/0.1-2.tif]

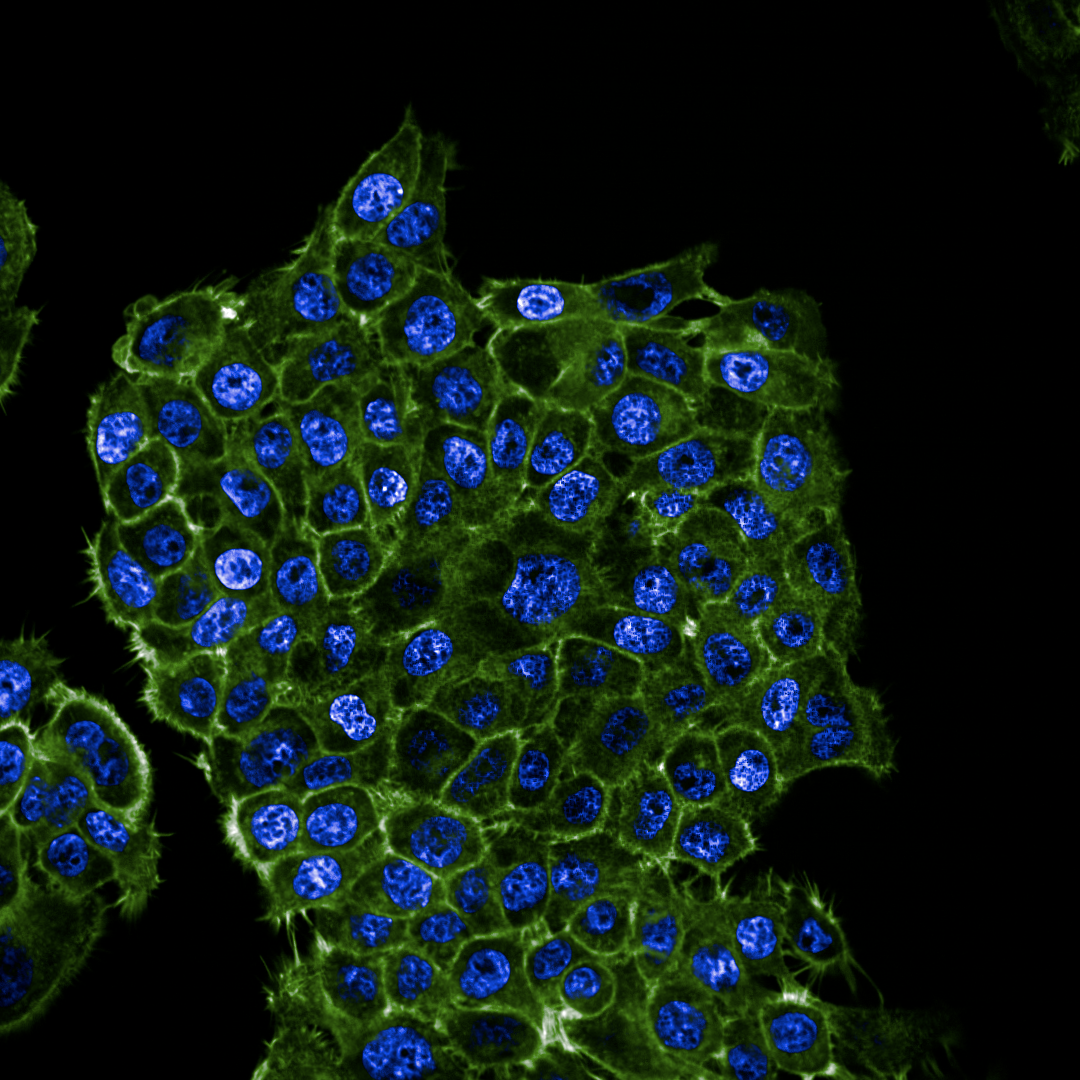

Supplement: Supplementary file 4 [file DataSheet1.ZIP › Figure 1-raw data/16HBE/0 mg∩╝Åml/0-1.png]

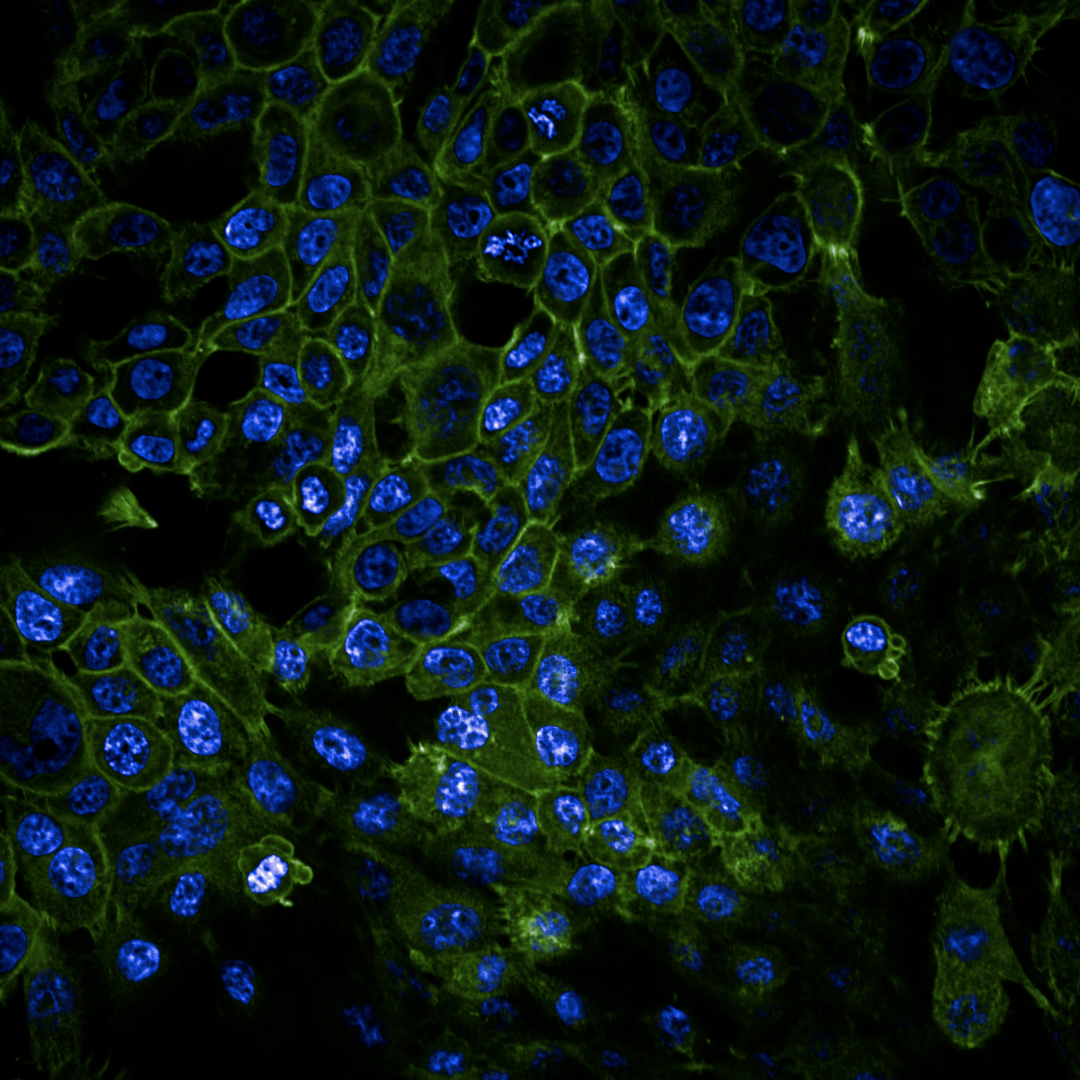

Supplement: Supplementary file 4 [file DataSheet1.ZIP › Figure 1-raw data/16HBE/0 mg∩╝Åml/0-2.png]

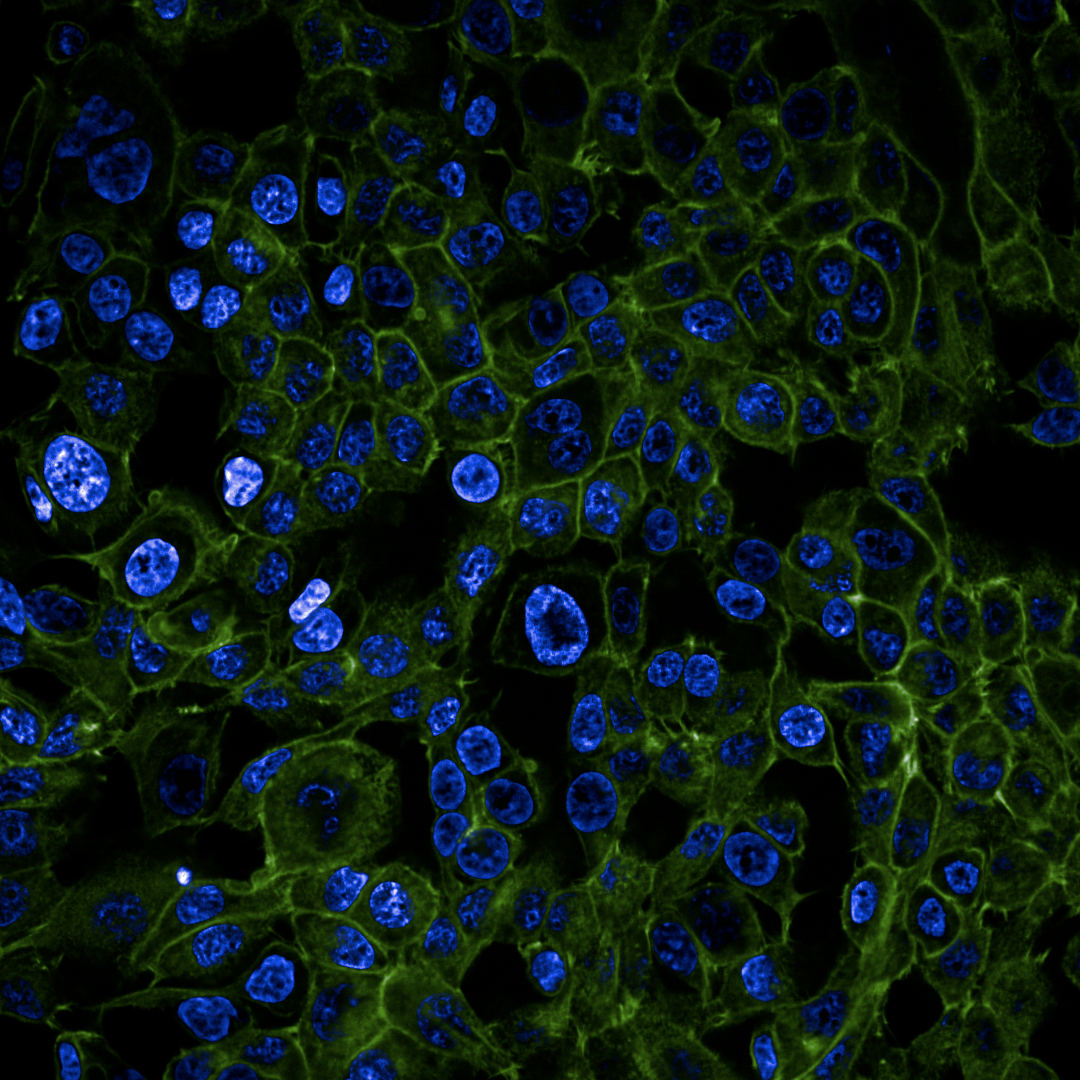

Supplement: Supplementary file 4 [file DataSheet1.ZIP › Figure 1-raw data/16HBE/0 mg∩╝Åml/0-3.png]

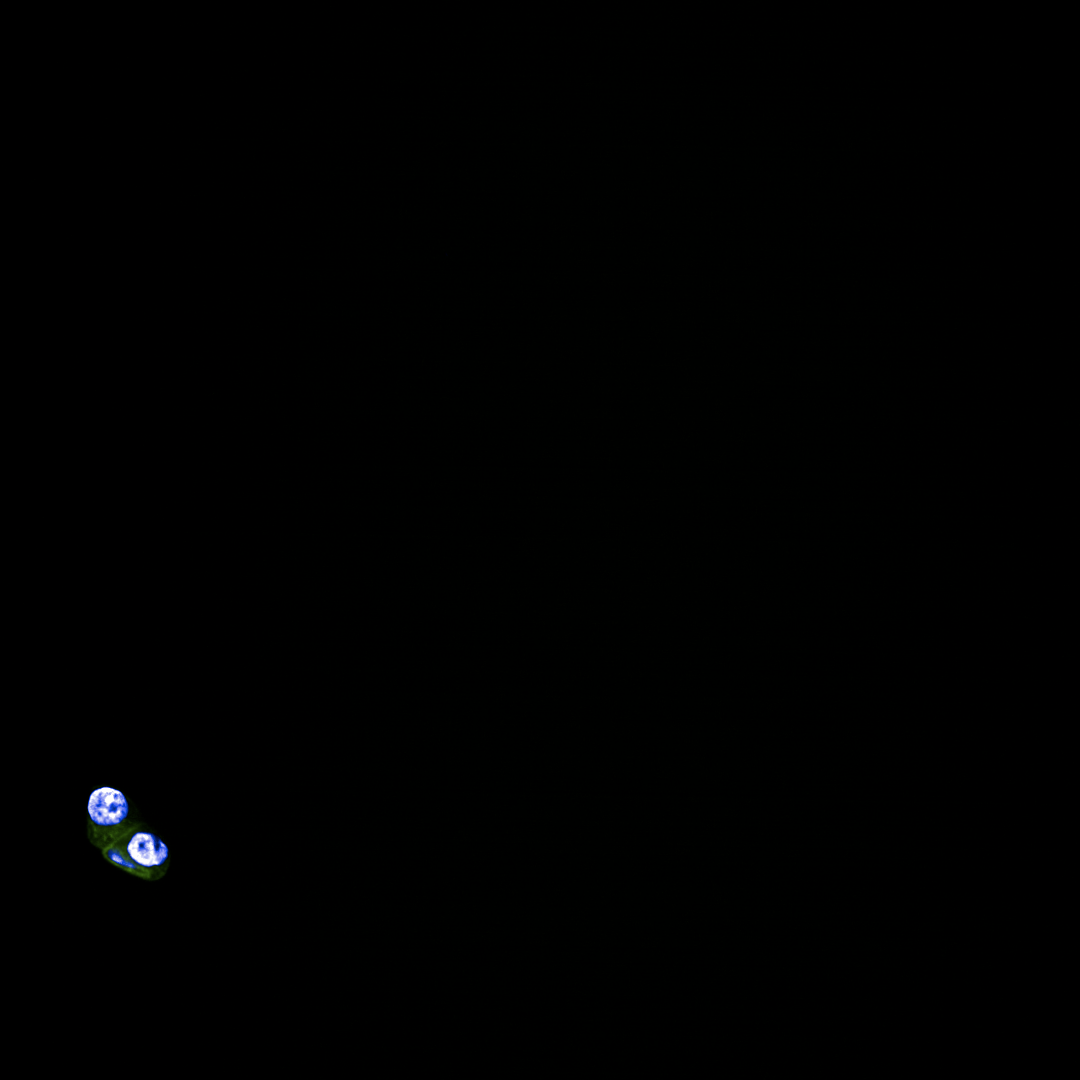

Supplement: Supplementary file 4 [file DataSheet1.ZIP › Figure 1-raw data/16HBE/100mg∩╝Åml/100-1.png]

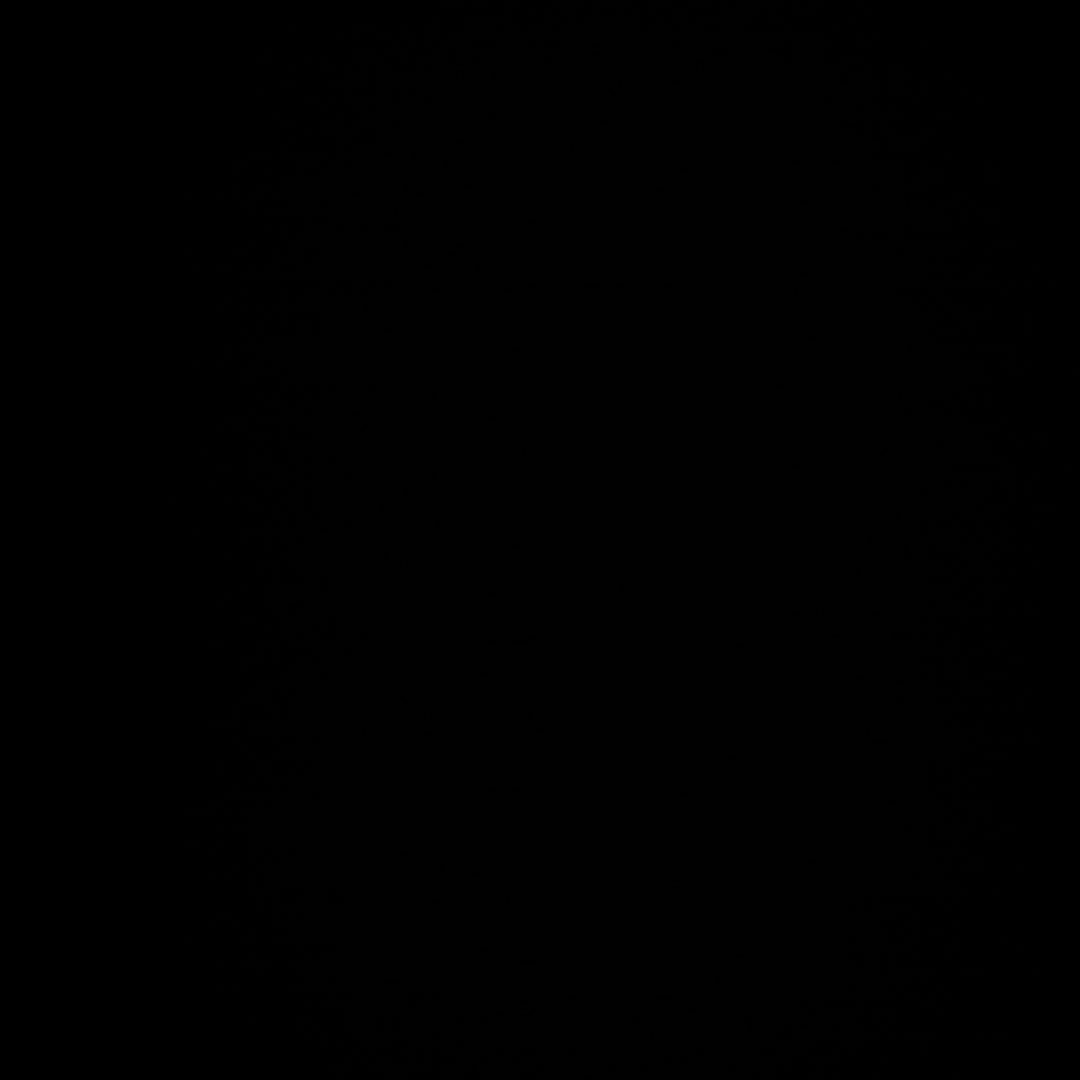

Supplement: Supplementary file 4 [file DataSheet1.ZIP › Figure 1-raw data/16HBE/100mg∩╝Åml/100-2.png]

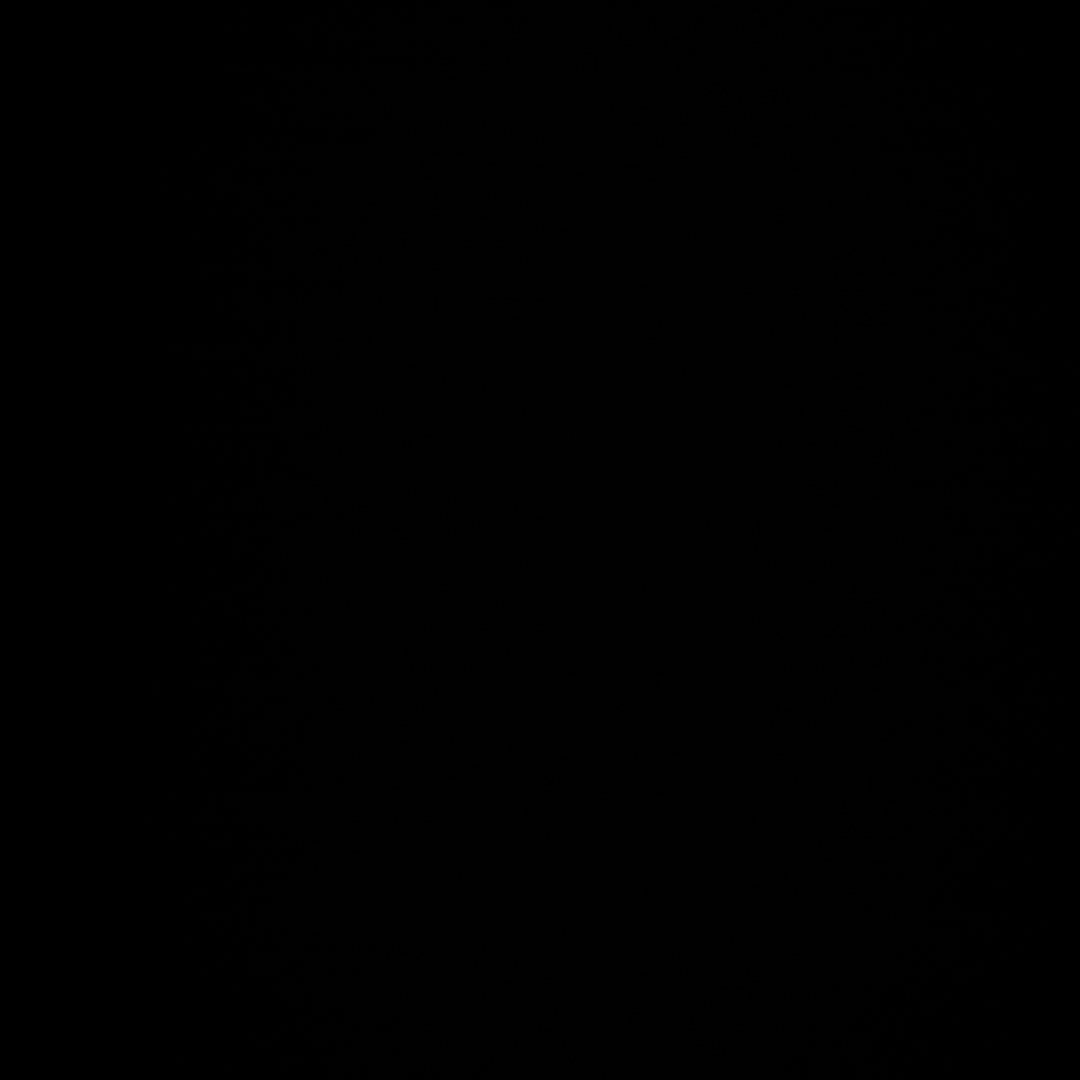

Supplement: Supplementary file 4 [file DataSheet1.ZIP › Figure 1-raw data/16HBE/100mg∩╝Åml/100-3.png]

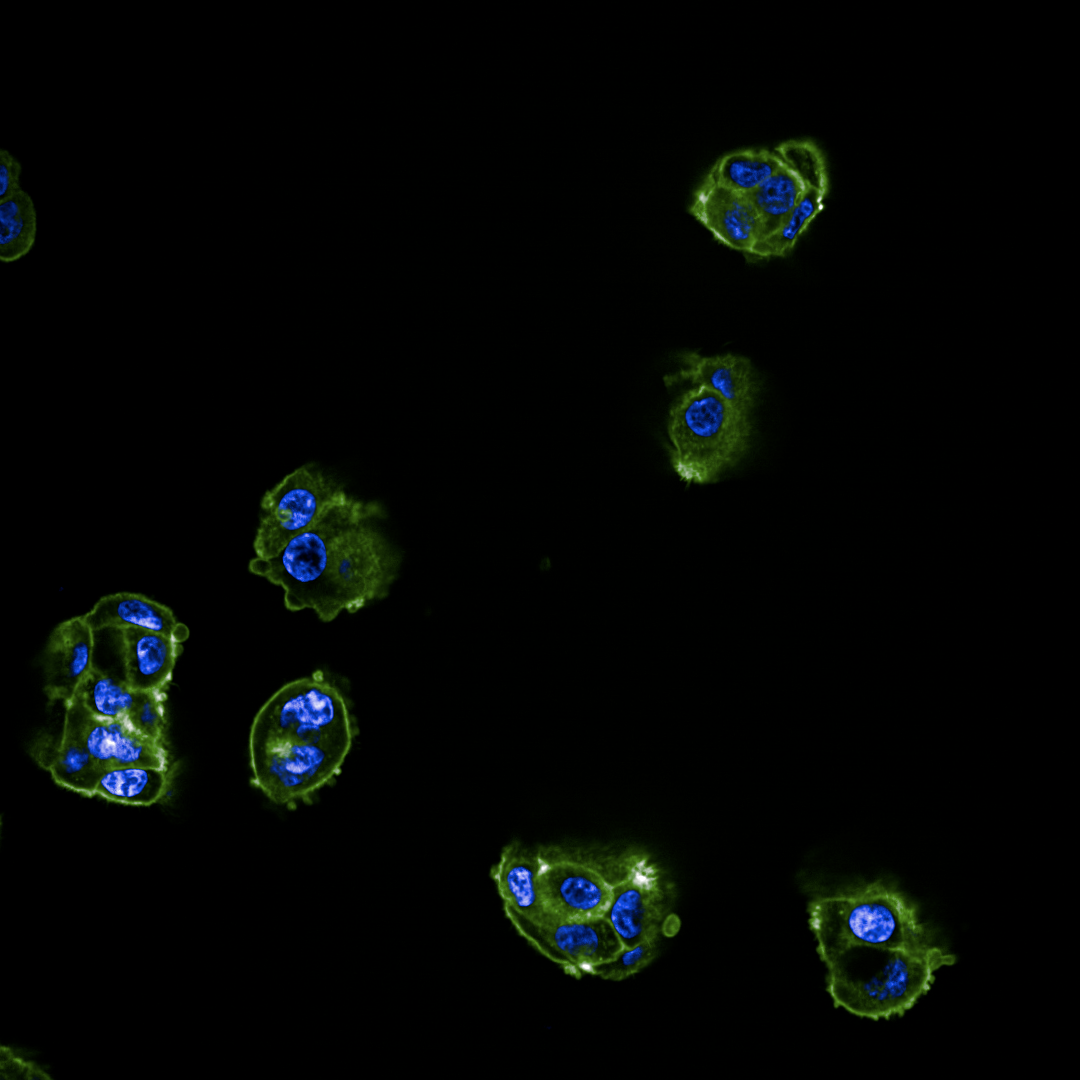

Supplement: Supplementary file 4 [file DataSheet1.ZIP › Figure 1-raw data/16HBE/10mg∩╝Åml/10-1.png]

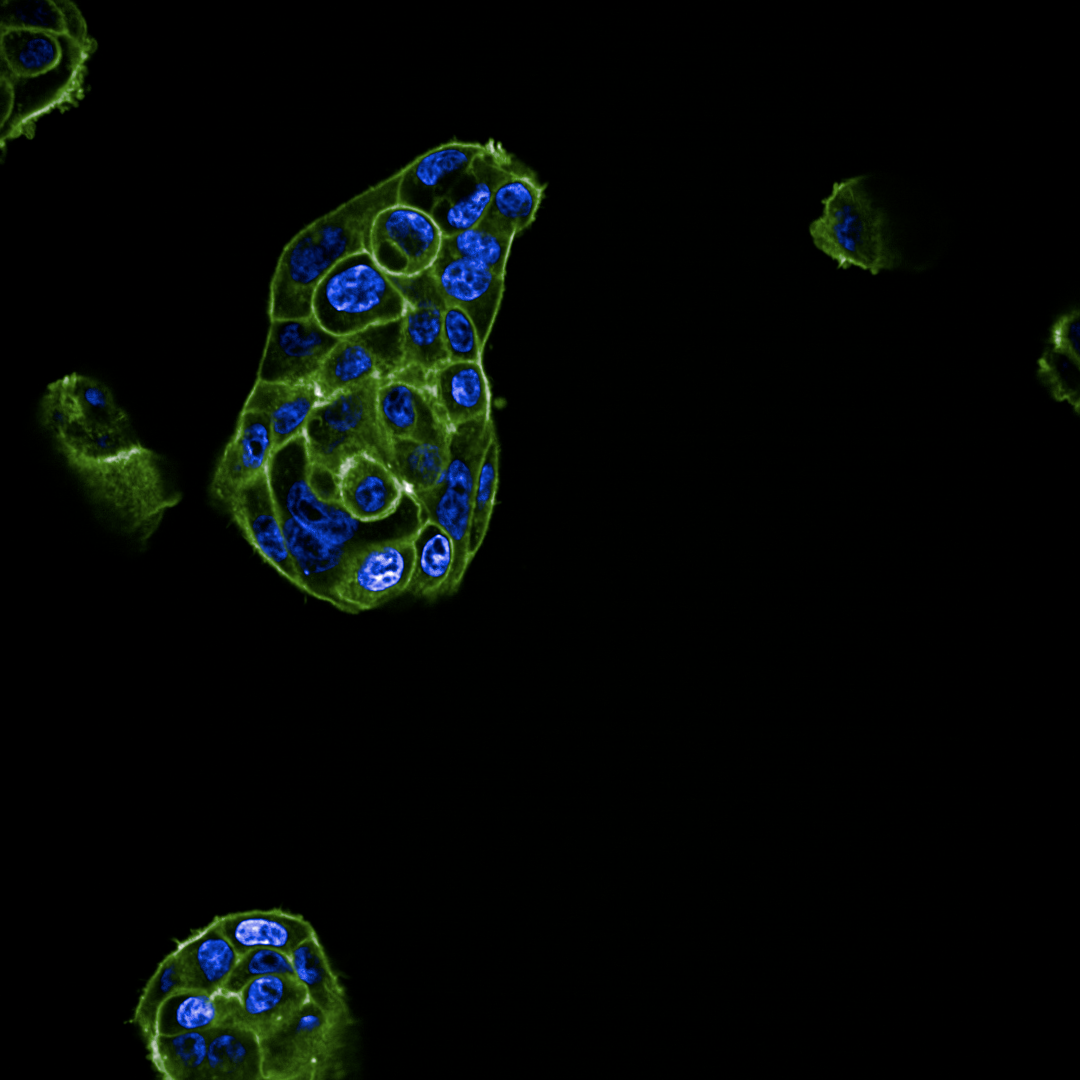

Supplement: Supplementary file 4 [file DataSheet1.ZIP › Figure 1-raw data/16HBE/10mg∩╝Åml/10-2.png]

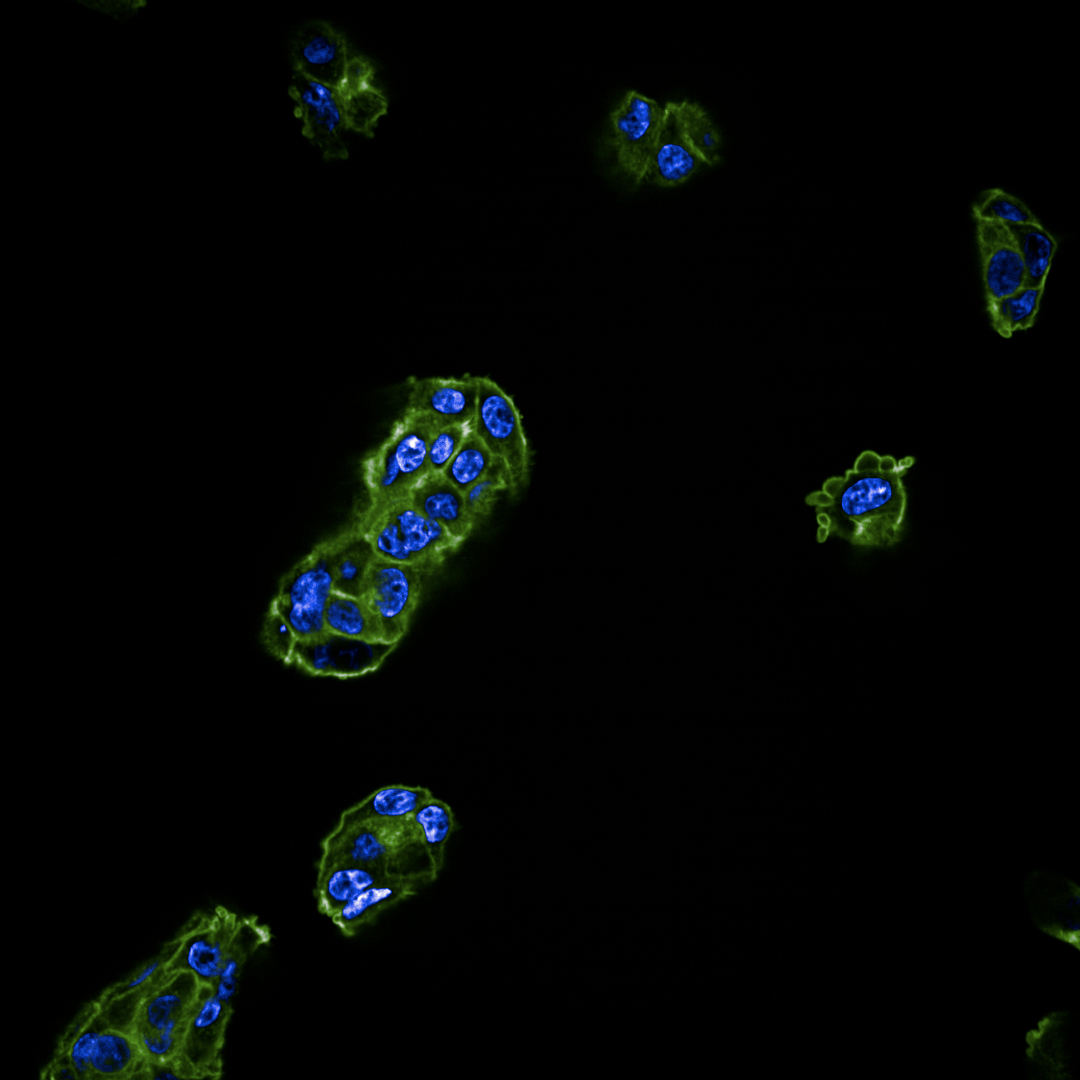

Supplement: Supplementary file 4 [file DataSheet1.ZIP › Figure 1-raw data/16HBE/10mg∩╝Åml/10-3.png]

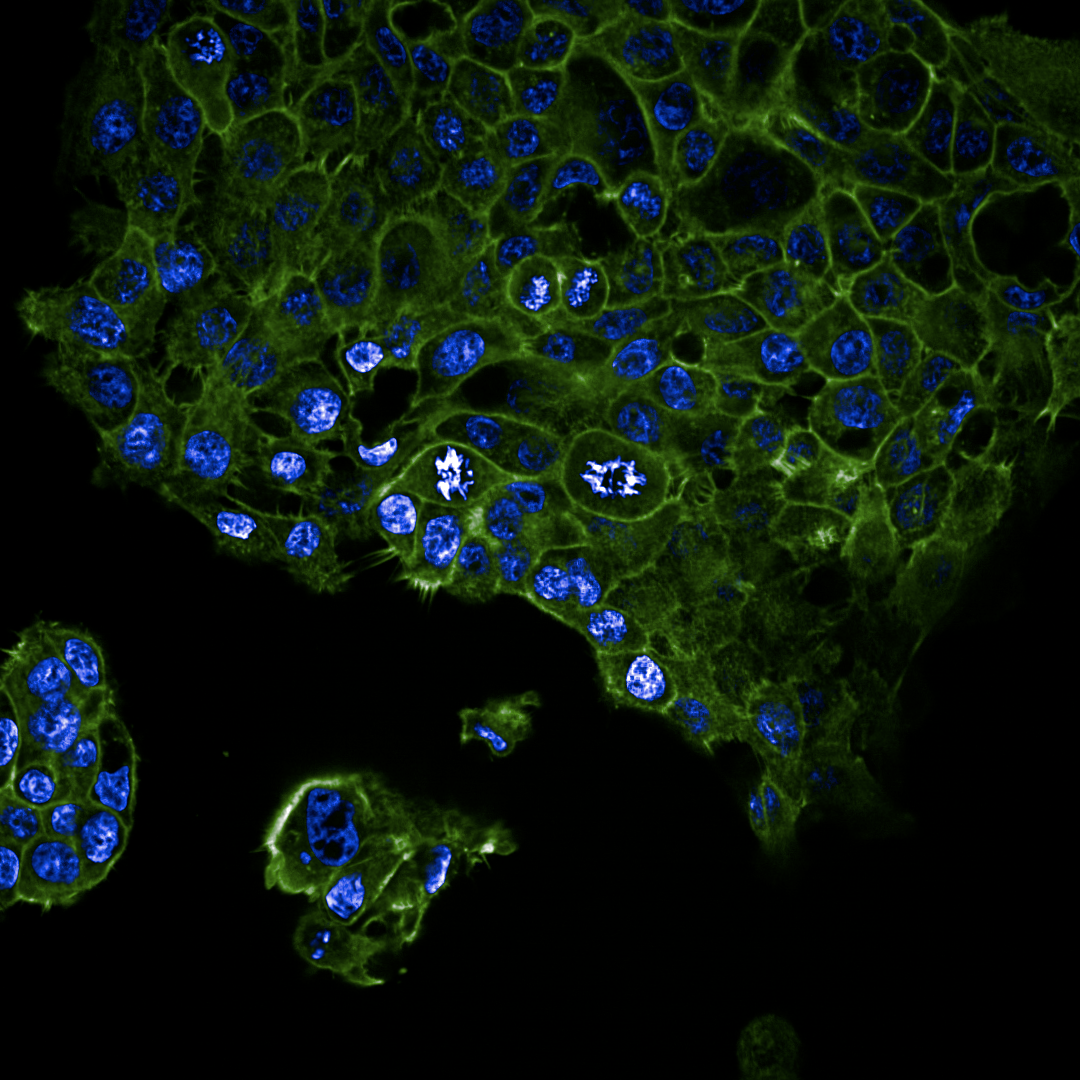

Supplement: Supplementary file 4 [file DataSheet1.ZIP › Figure 1-raw data/16HBE/1mg∩╝Åml/1-1.png]

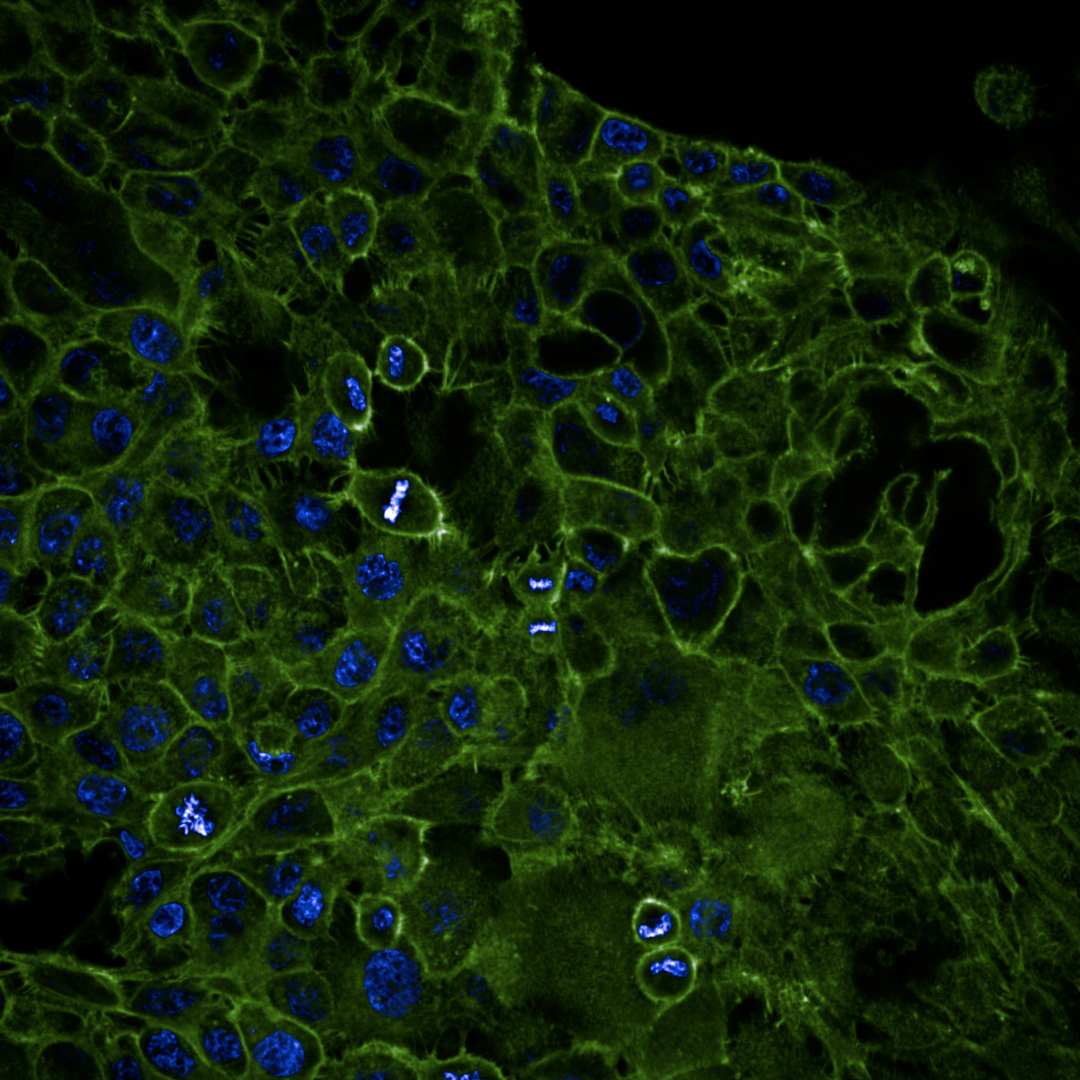

Supplement: Supplementary file 4 [file DataSheet1.ZIP › Figure 1-raw data/16HBE/1mg∩╝Åml/1-2.png]

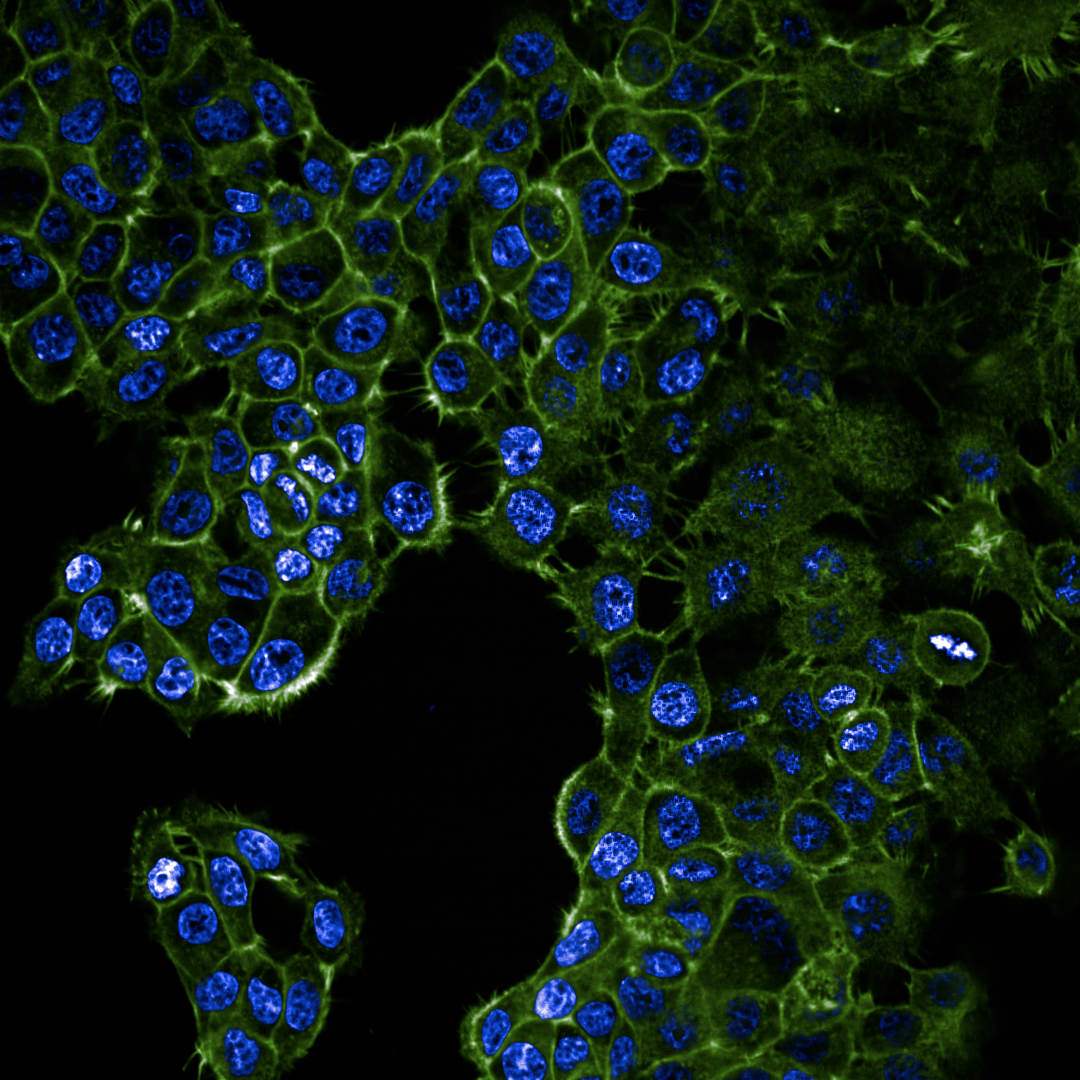

Supplement: Supplementary file 4 [file DataSheet1.ZIP › Figure 1-raw data/16HBE/1mg∩╝Åml/1-3.png]

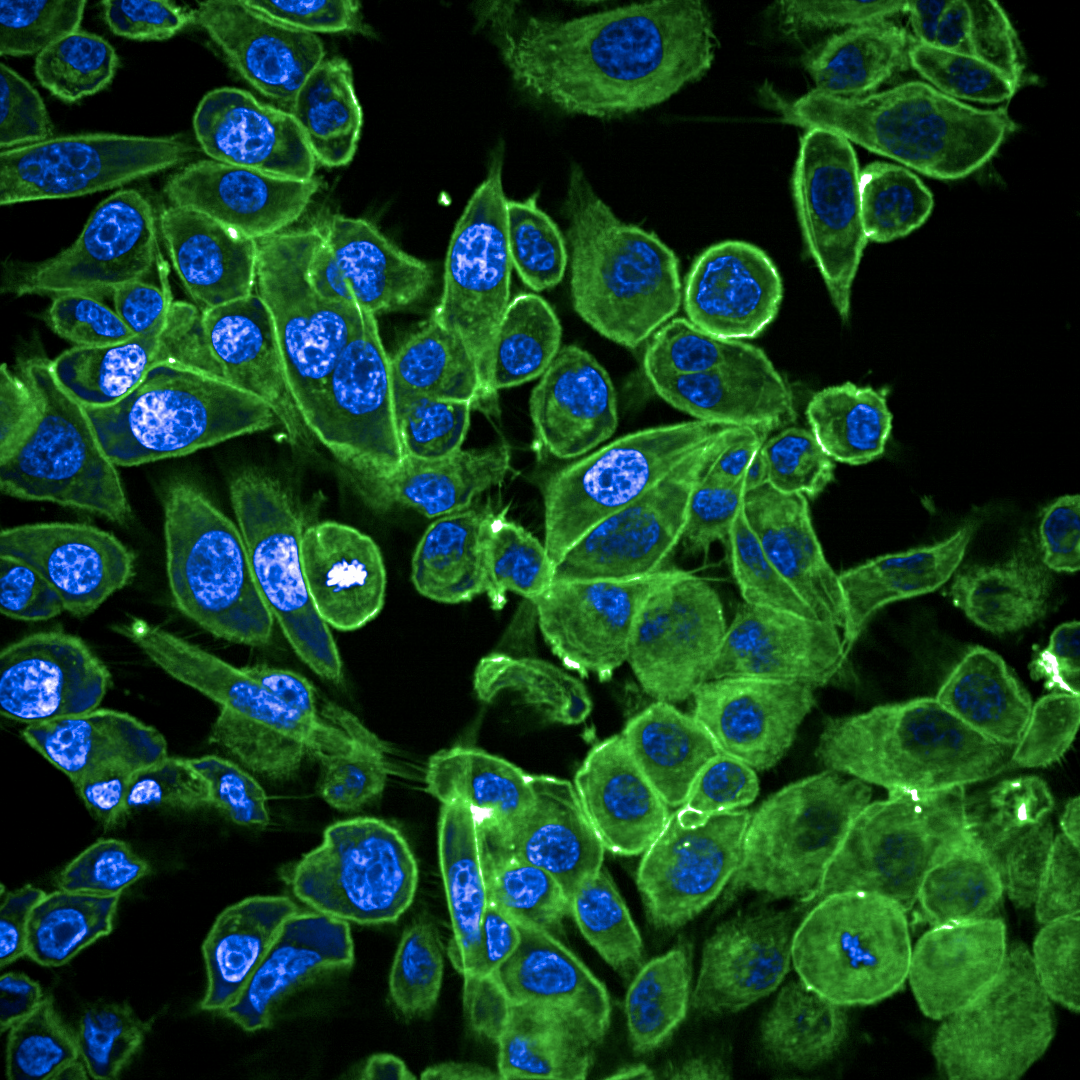

Supplement: Supplementary file 4 [file DataSheet1.ZIP › Figure 1-raw data/H226/0mg:ml/0-1.png]

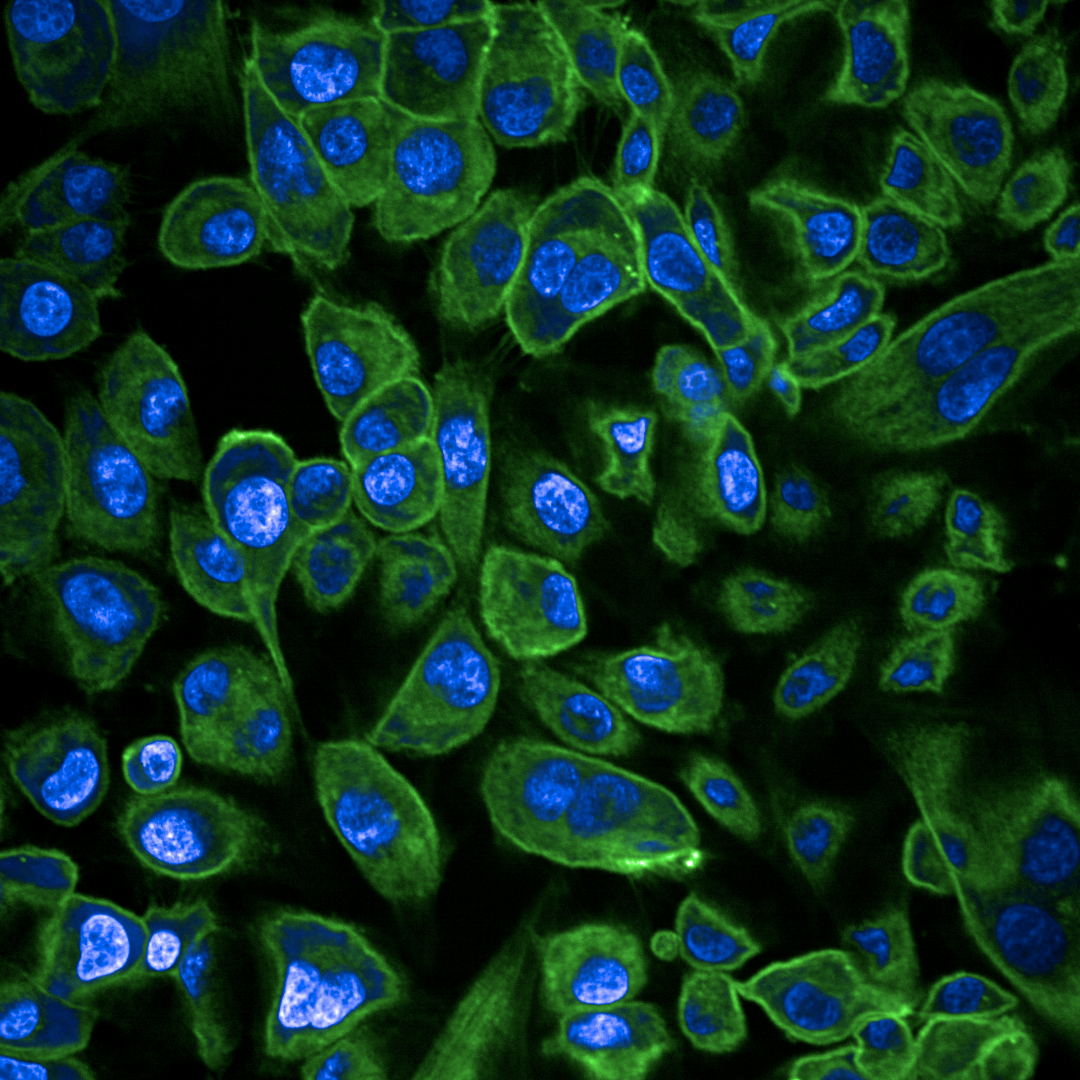

Supplement: Supplementary file 4 [file DataSheet1.ZIP › Figure 1-raw data/H226/0mg:ml/0-2.png]

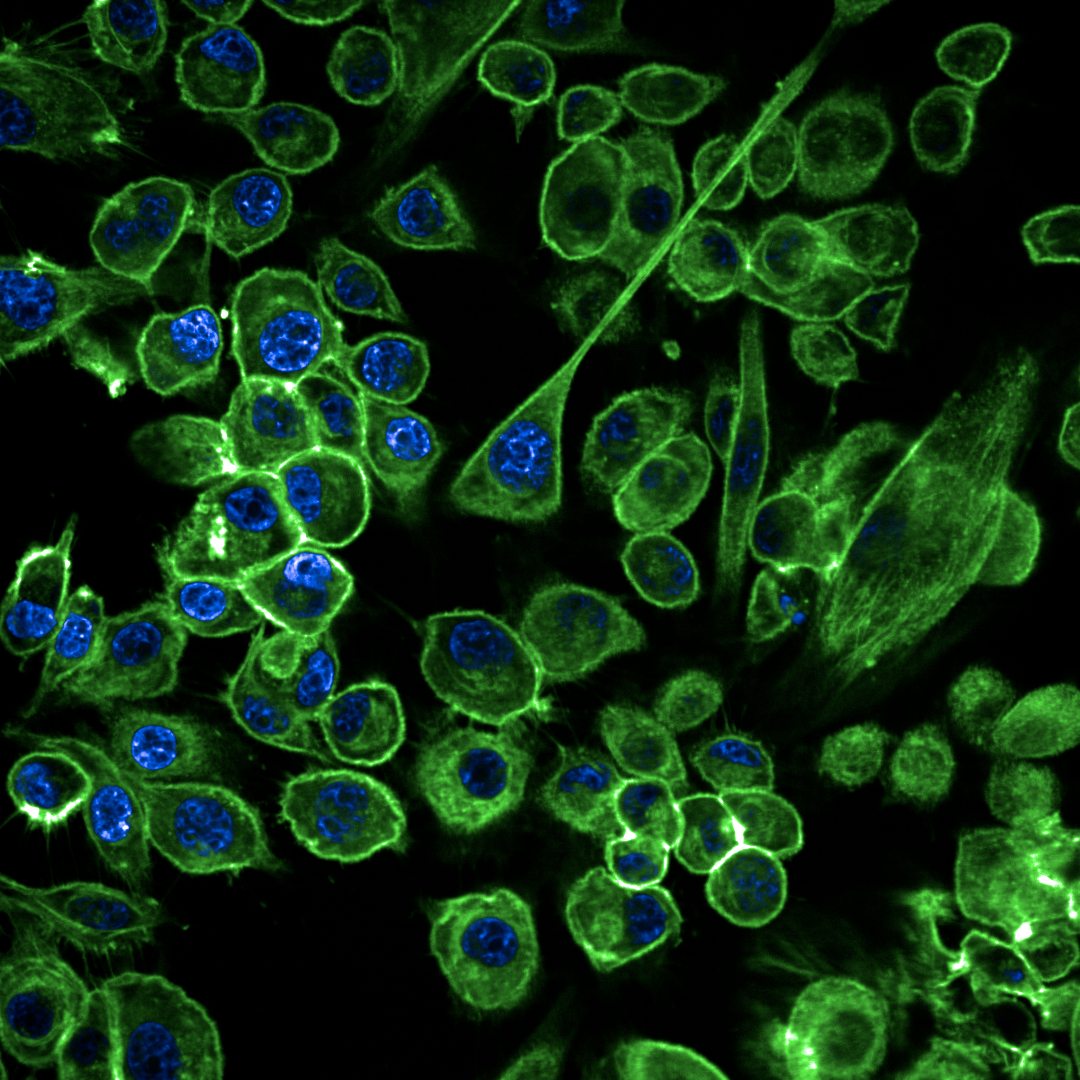

Supplement: Supplementary file 4 [file DataSheet1.ZIP › Figure 1-raw data/H226/0mg:ml/0-3.png]

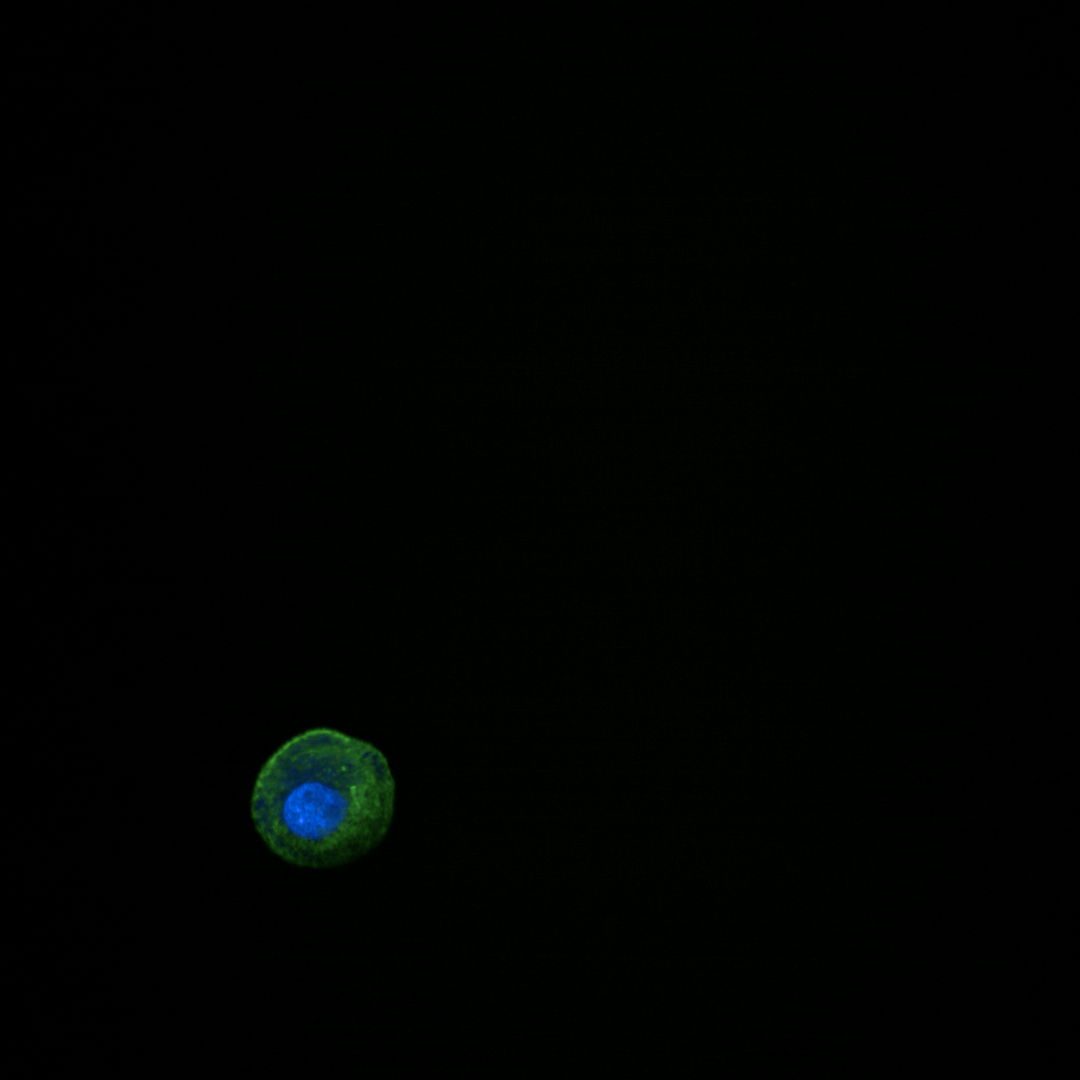

Supplement: Supplementary file 4 [file DataSheet1.ZIP › Figure 1-raw data/H226/100mg:ml/100-1.png]

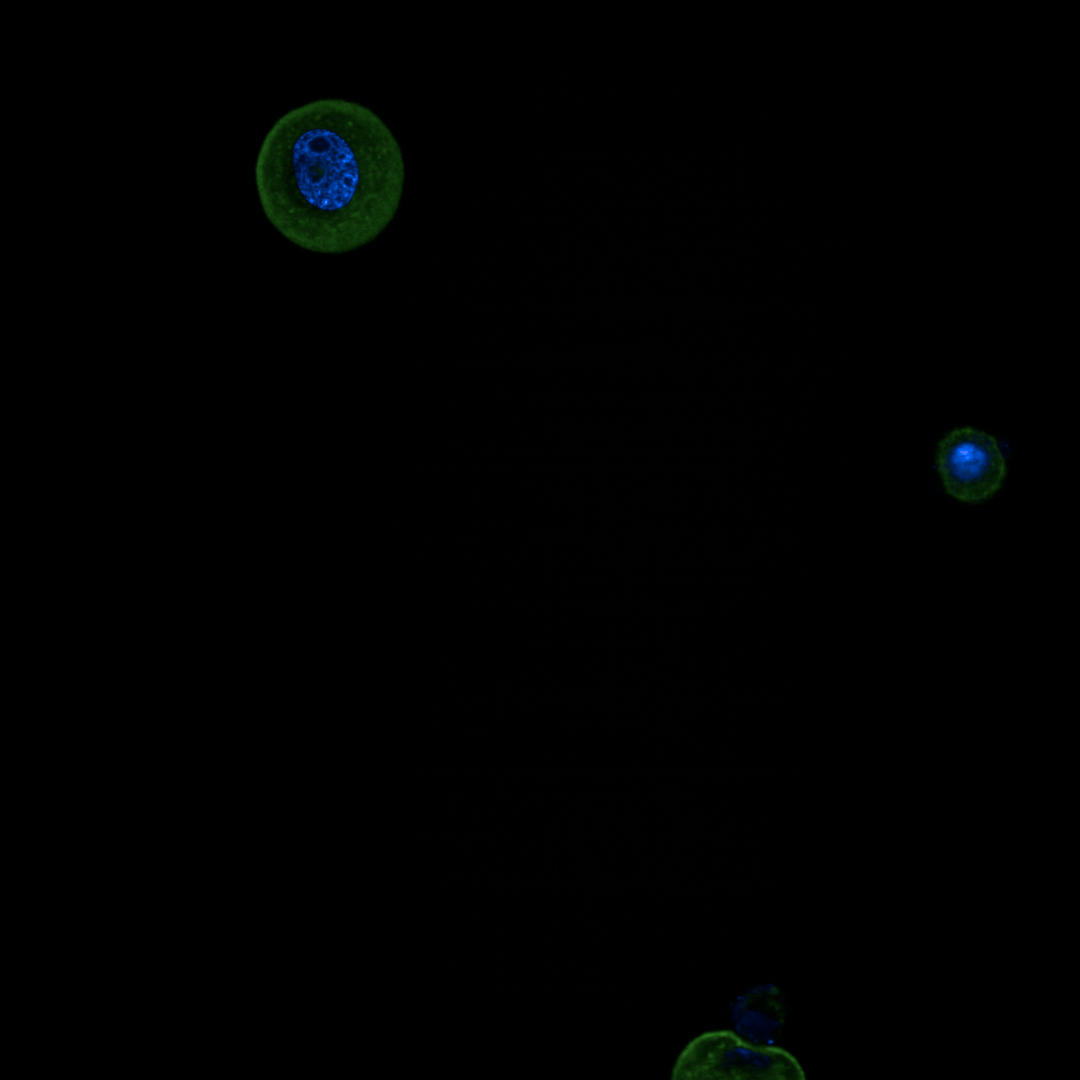

Supplement: Supplementary file 4 [file DataSheet1.ZIP › Figure 1-raw data/H226/100mg:ml/100-2.png]

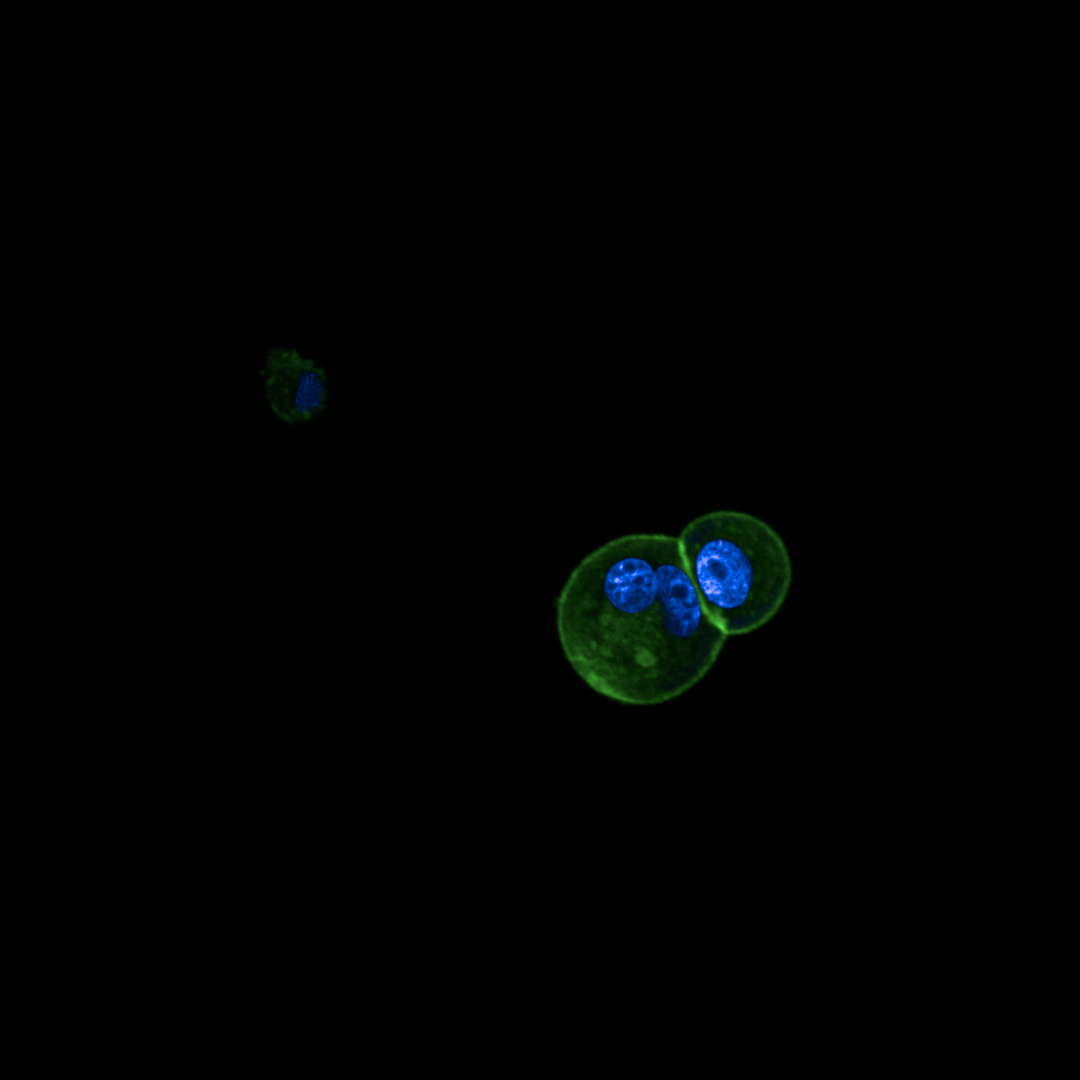

Supplement: Supplementary file 4 [file DataSheet1.ZIP › Figure 1-raw data/H226/100mg:ml/100-3.png]

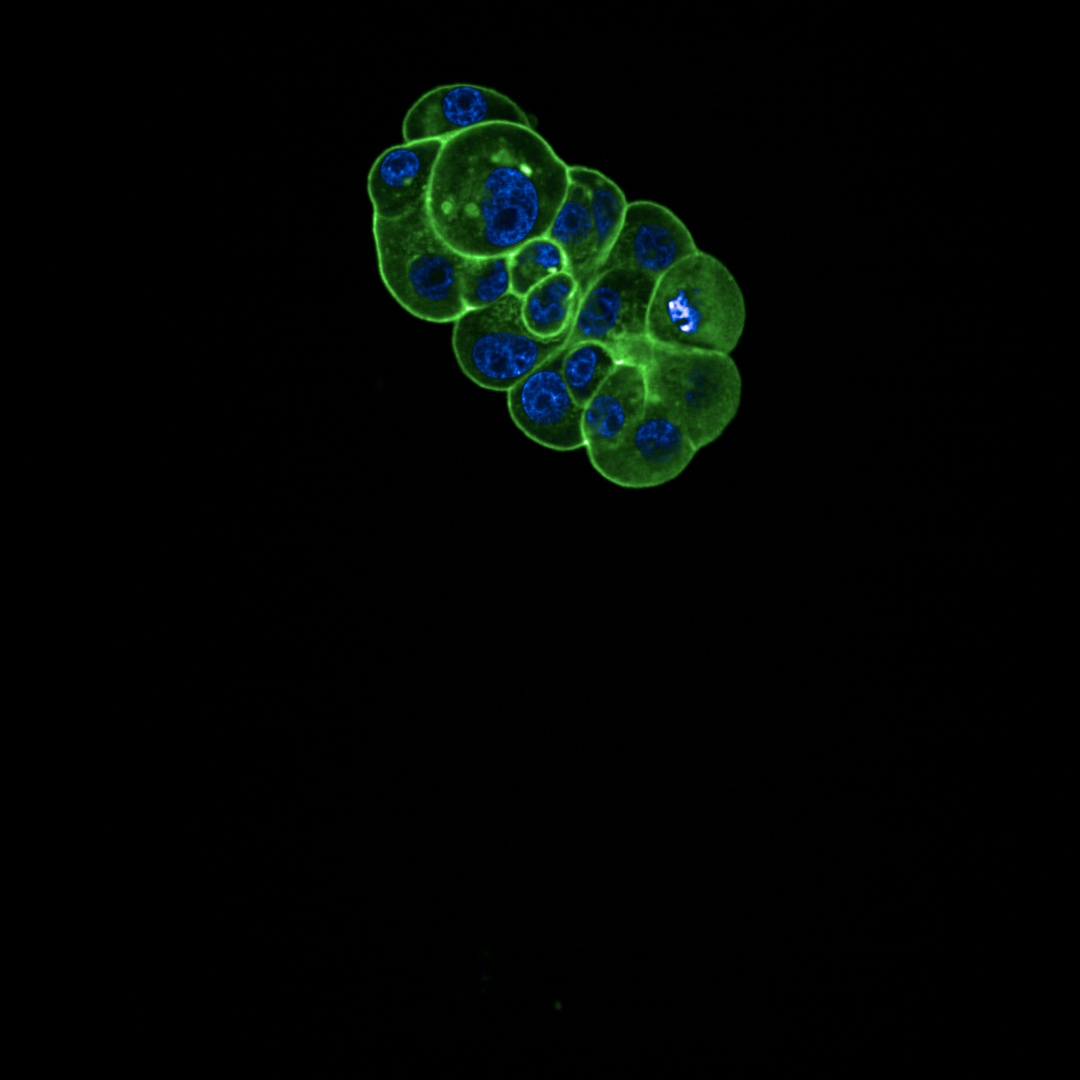

Supplement: Supplementary file 4 [file DataSheet1.ZIP › Figure 1-raw data/H226/10mg:ml/10-1.png]

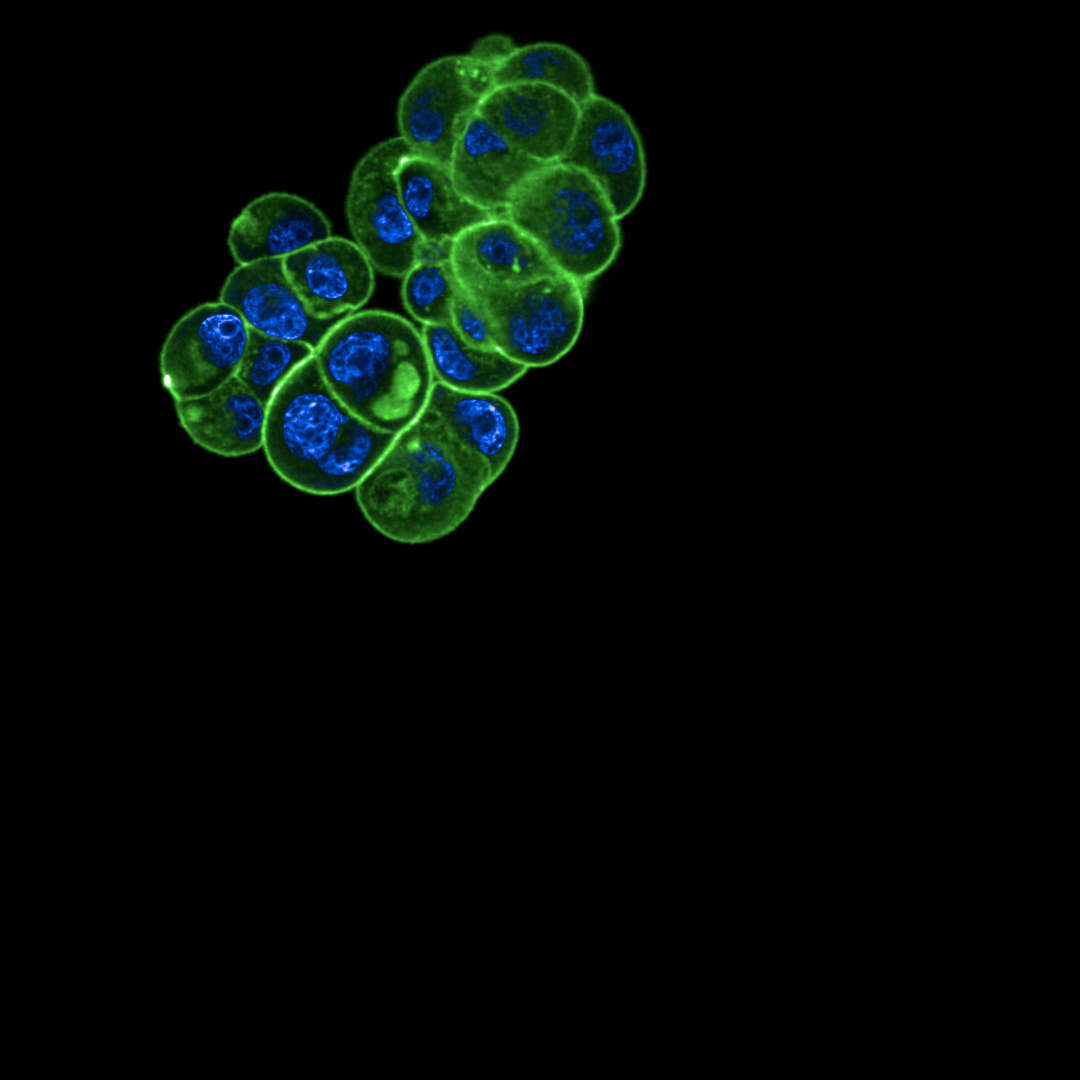

Supplement: Supplementary file 4 [file DataSheet1.ZIP › Figure 1-raw data/H226/10mg:ml/10-2.png]

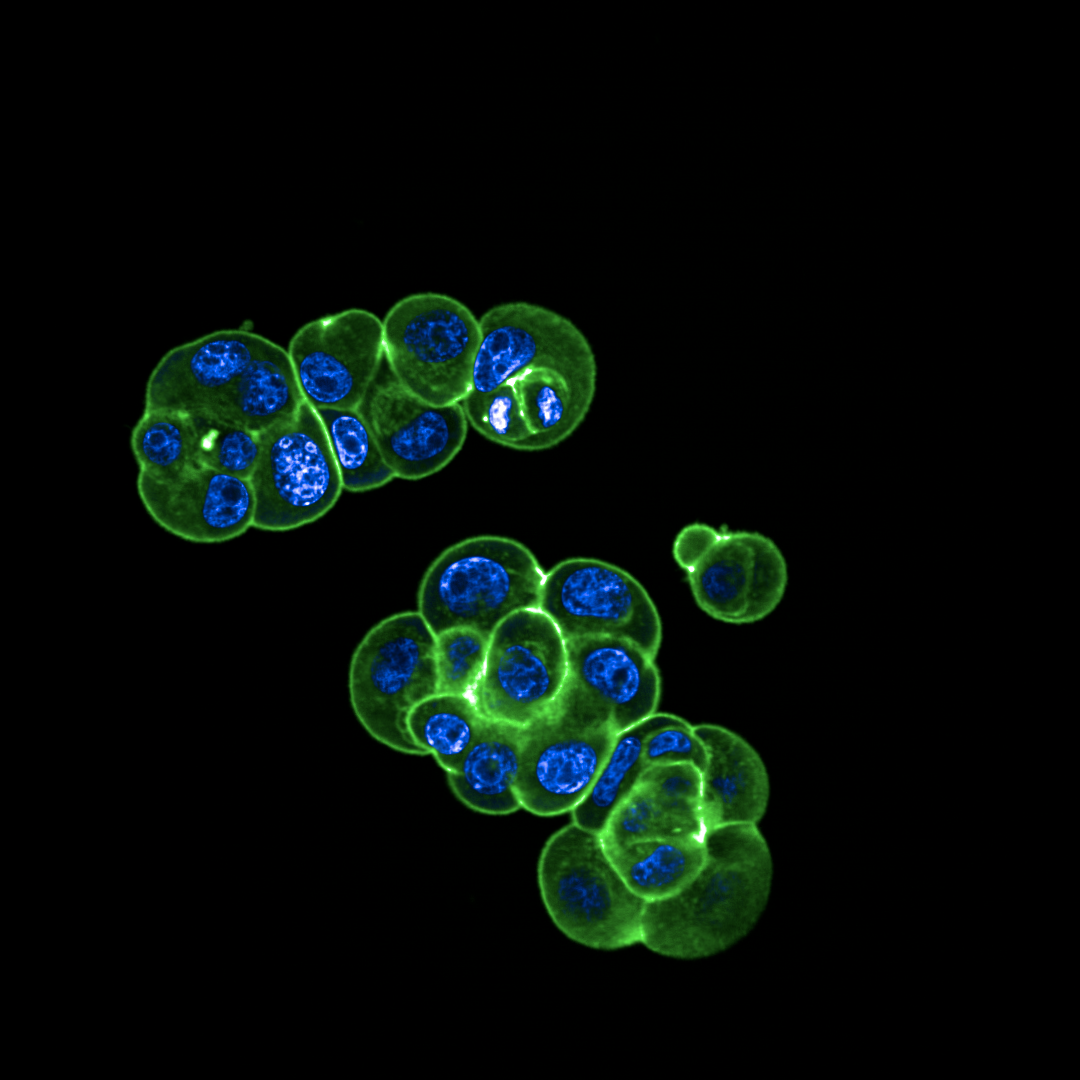

Supplement: Supplementary file 4 [file DataSheet1.ZIP › Figure 1-raw data/H226/10mg:ml/10-3.png]

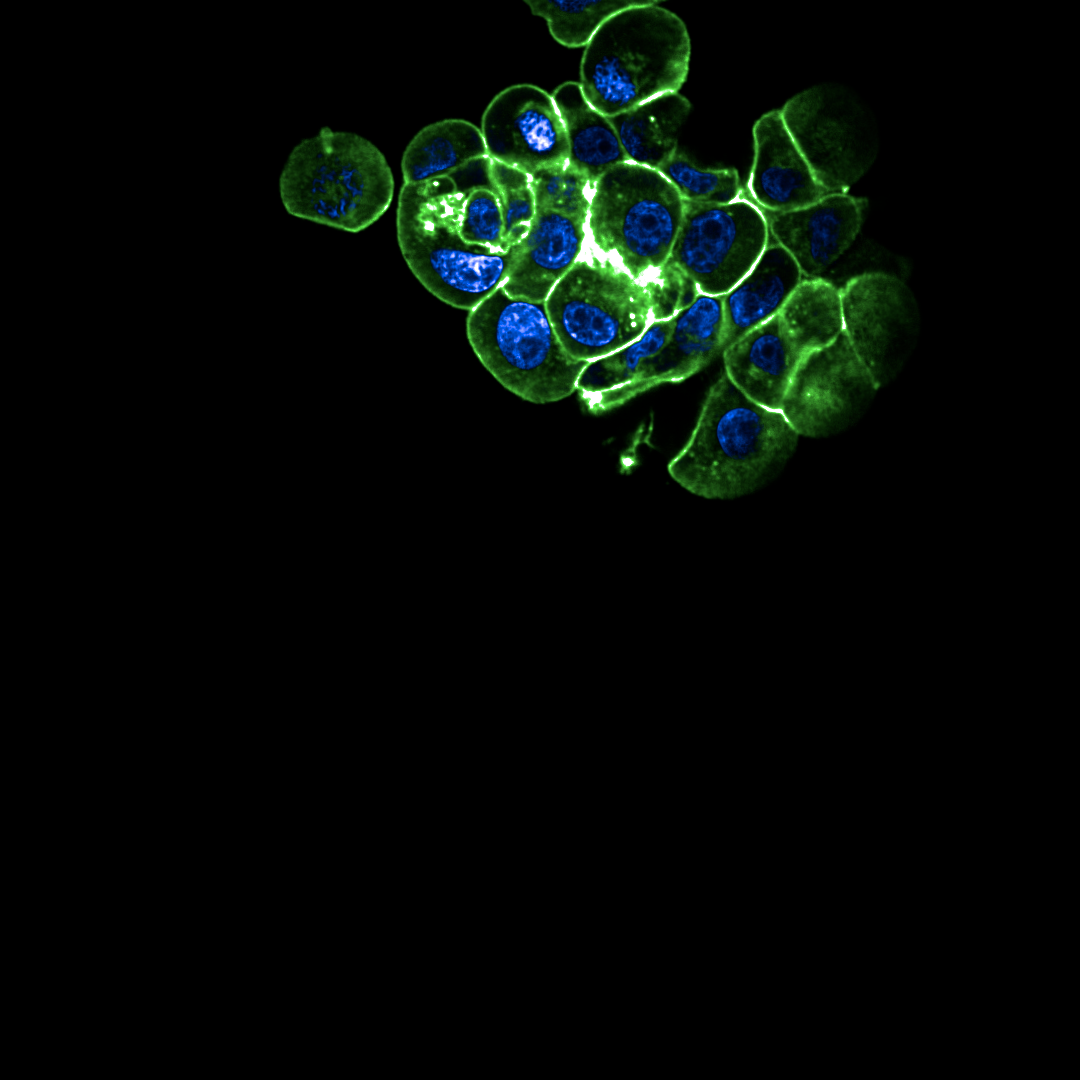

Supplement: Supplementary file 4 [file DataSheet1.ZIP › Figure 1-raw data/H226/1mg:ml/1-1.png]

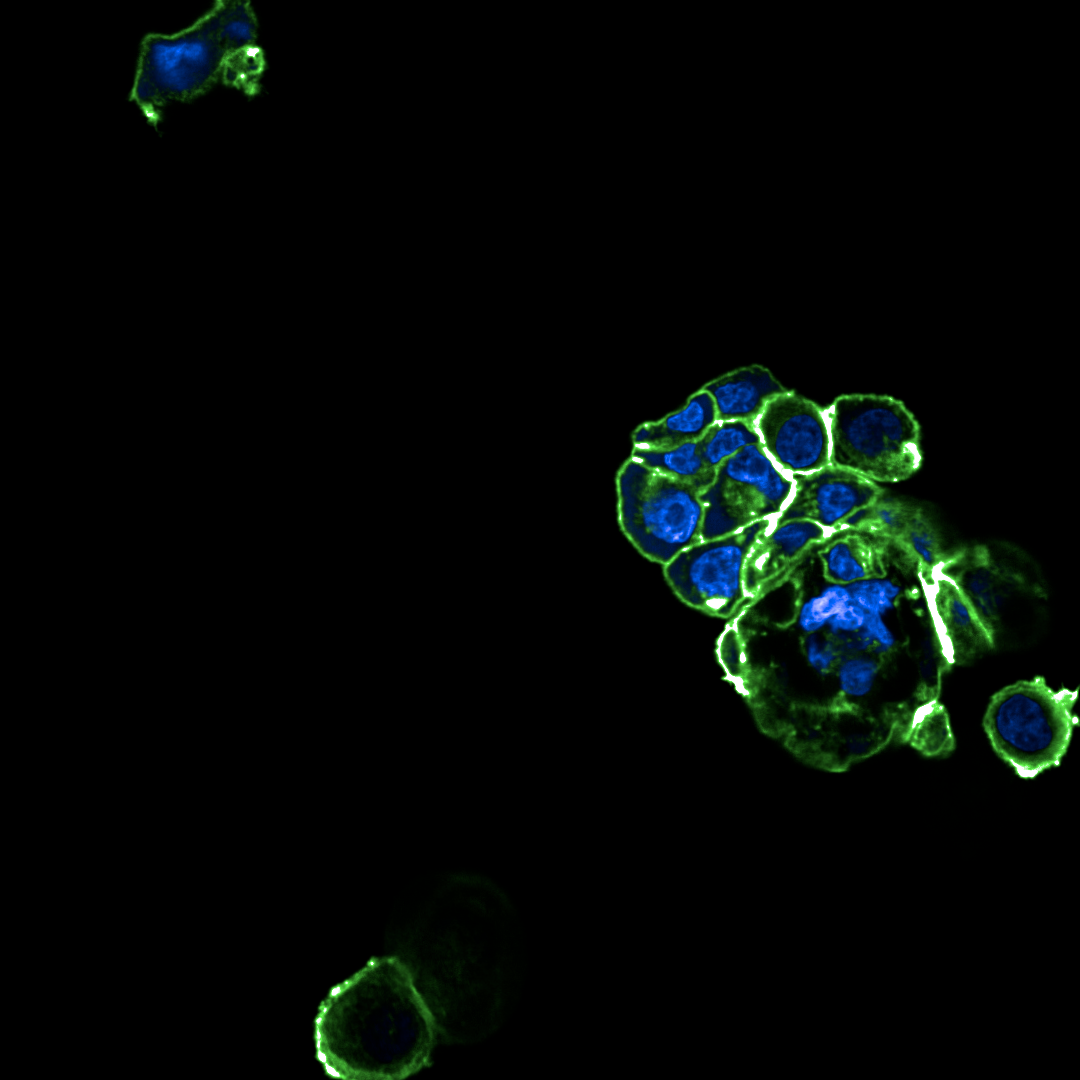

Supplement: Supplementary file 4 [file DataSheet1.ZIP › Figure 1-raw data/H226/1mg:ml/1-2.png]

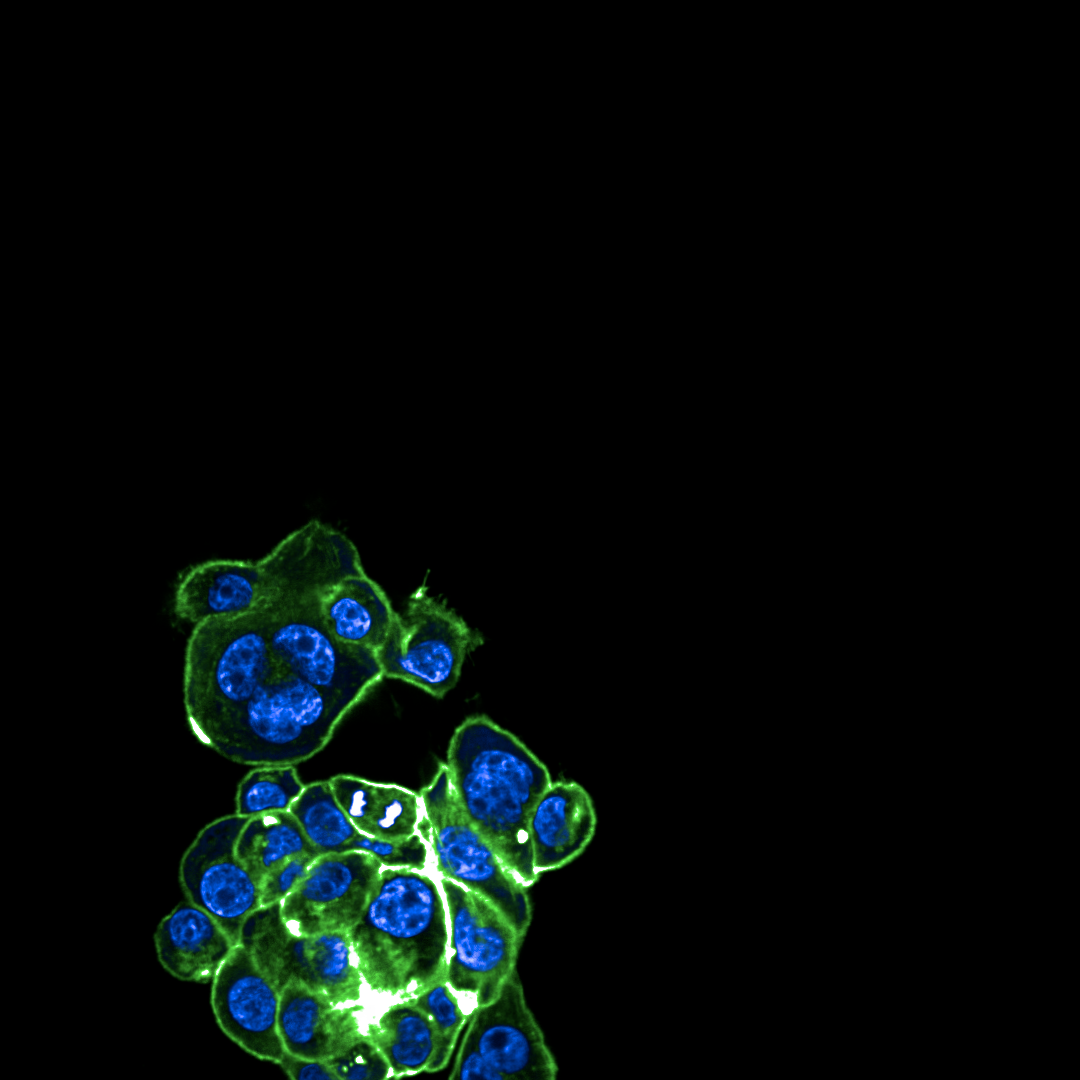

Supplement: Supplementary file 4 [file DataSheet1.ZIP › Figure 1-raw data/H226/1mg:ml/1-3.png]

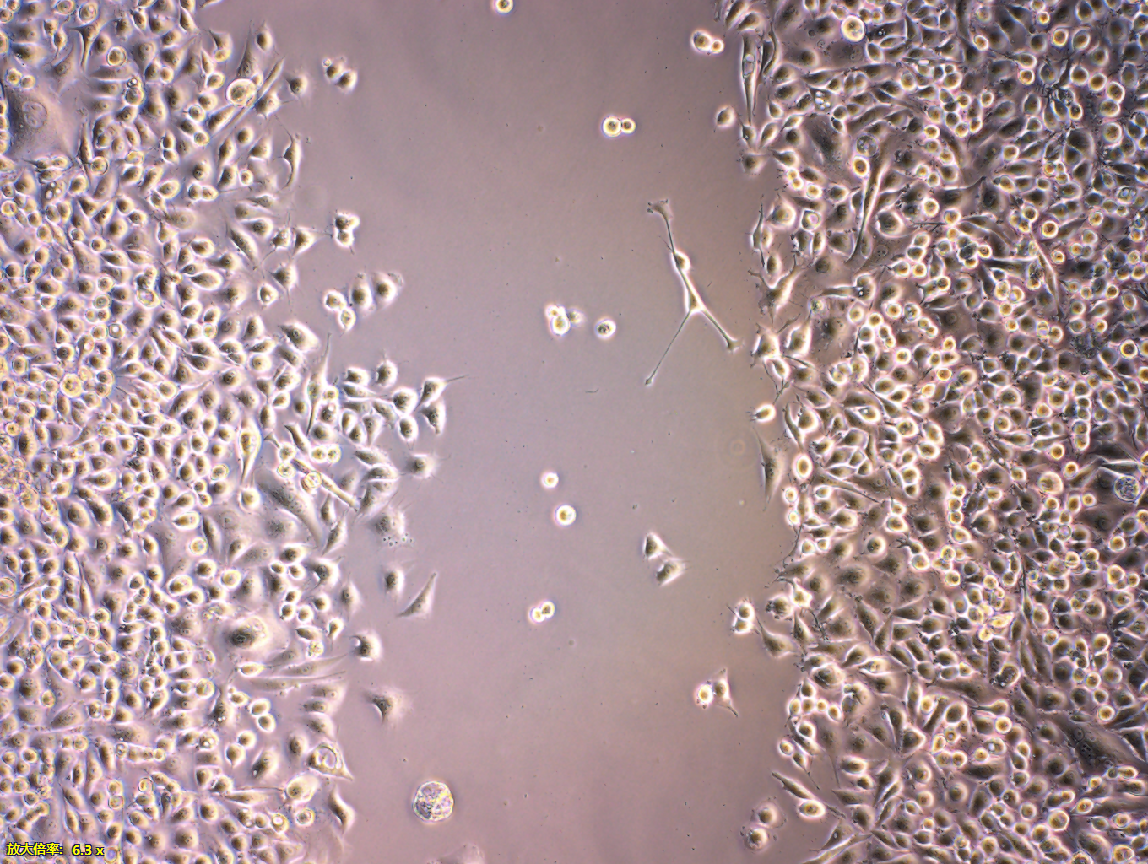

Supplement: Supplementary file 5 [file DataSheet6.ZIP › 48h/0-1.tif]

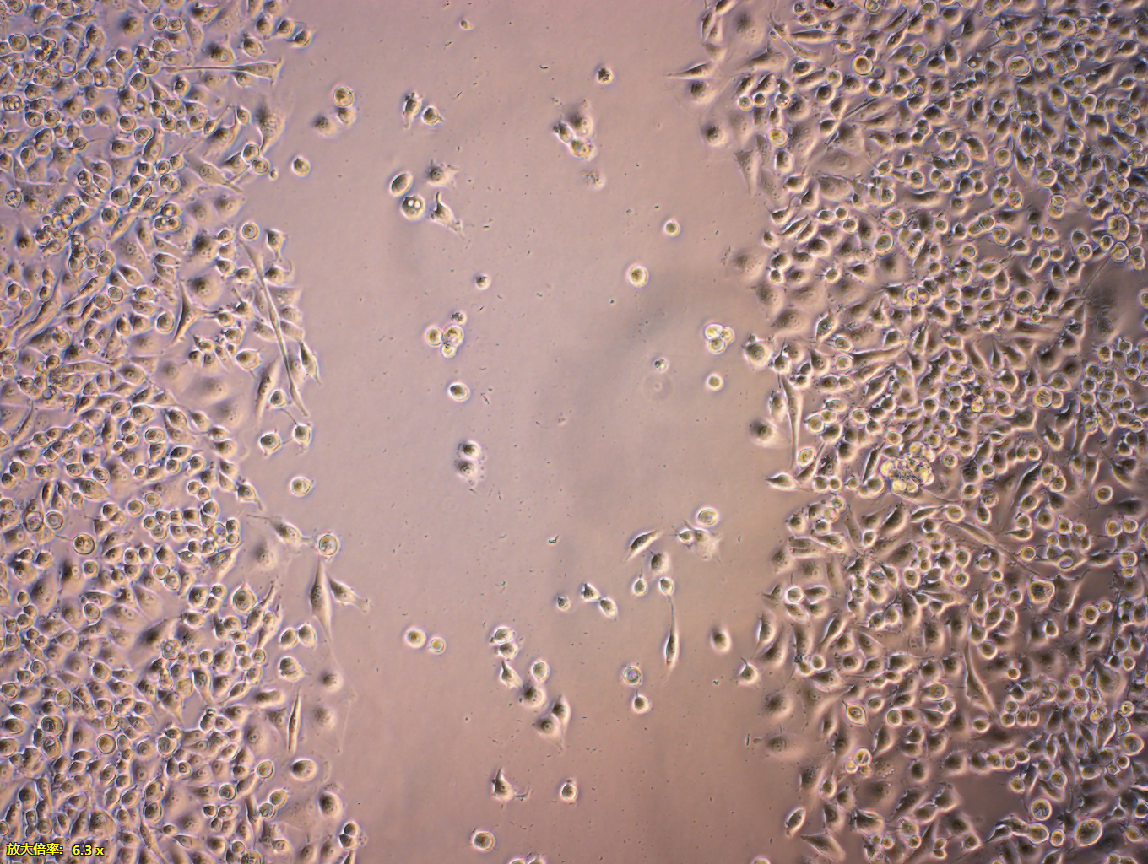

Supplement: Supplementary file 5 [file DataSheet6.ZIP › 48h/0-2.tif]

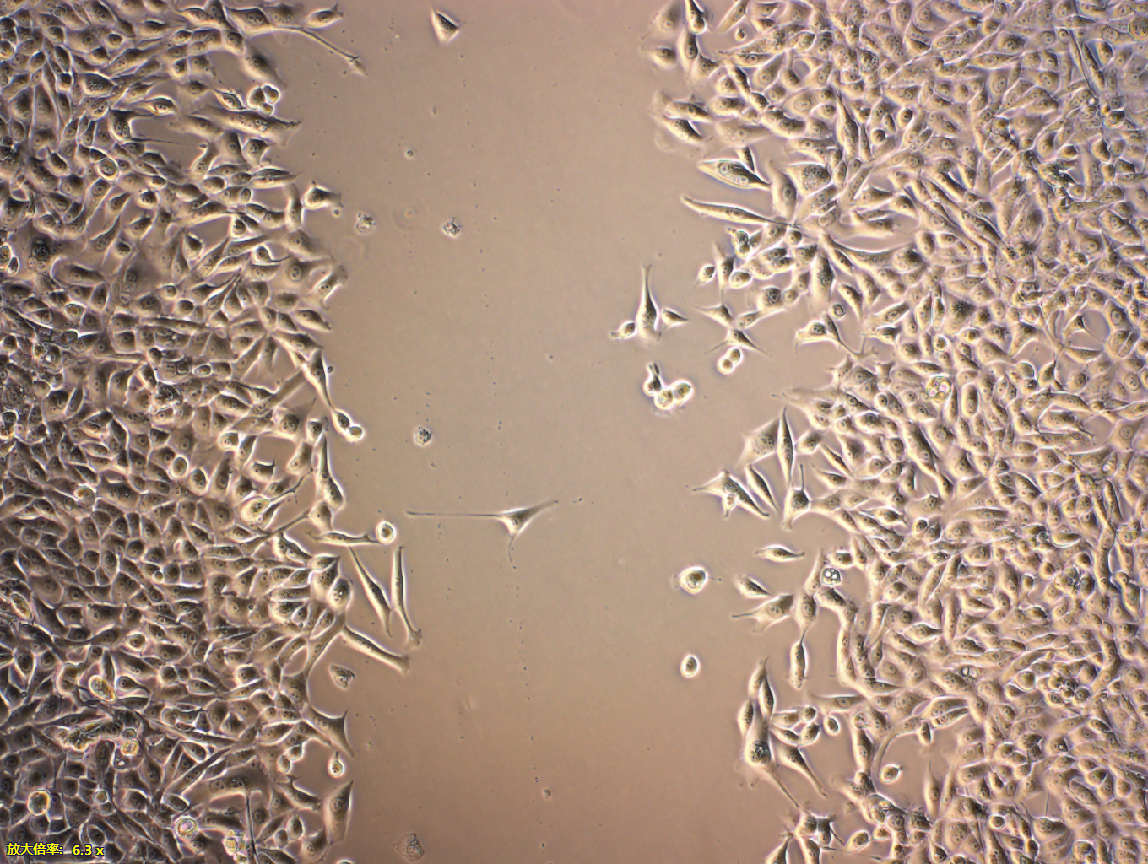

Supplement: Supplementary file 5 [file DataSheet6.ZIP › 48h/0.1-1.tif]

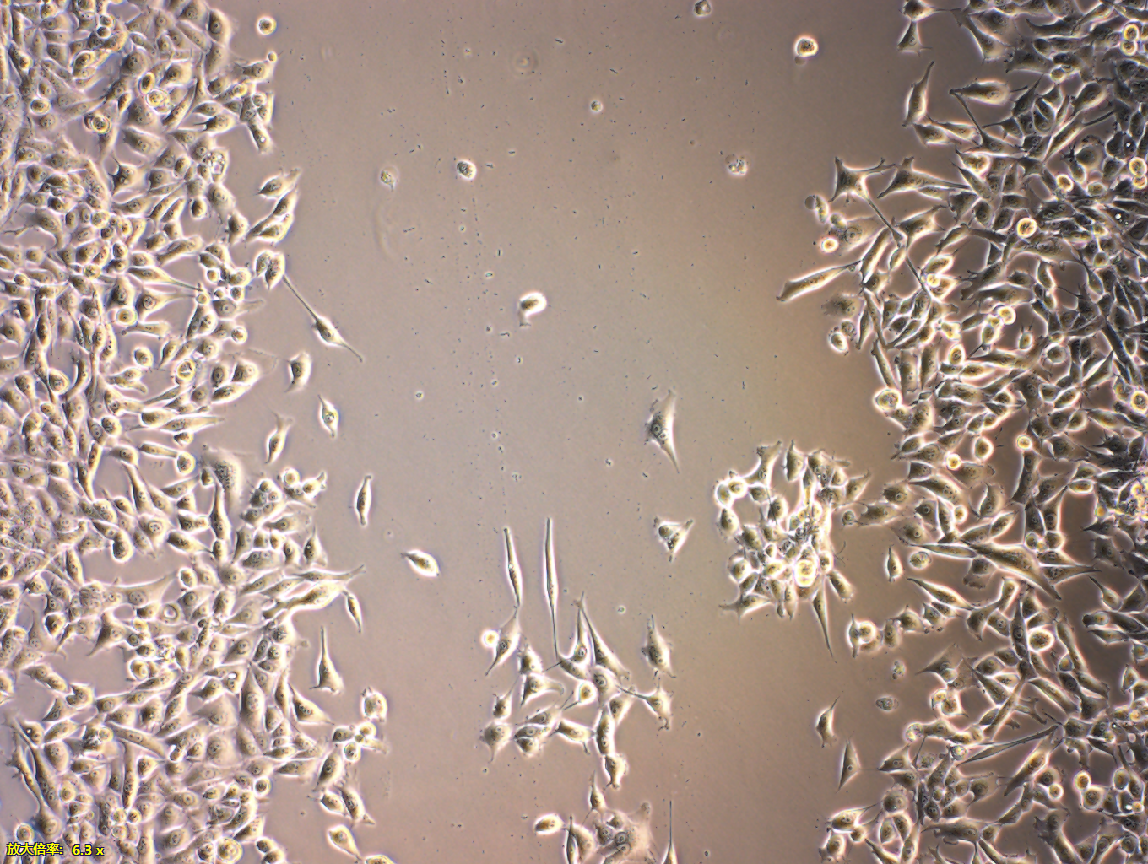

Supplement: Supplementary file 5 [file DataSheet6.ZIP › 48h/0.1-2.tif]

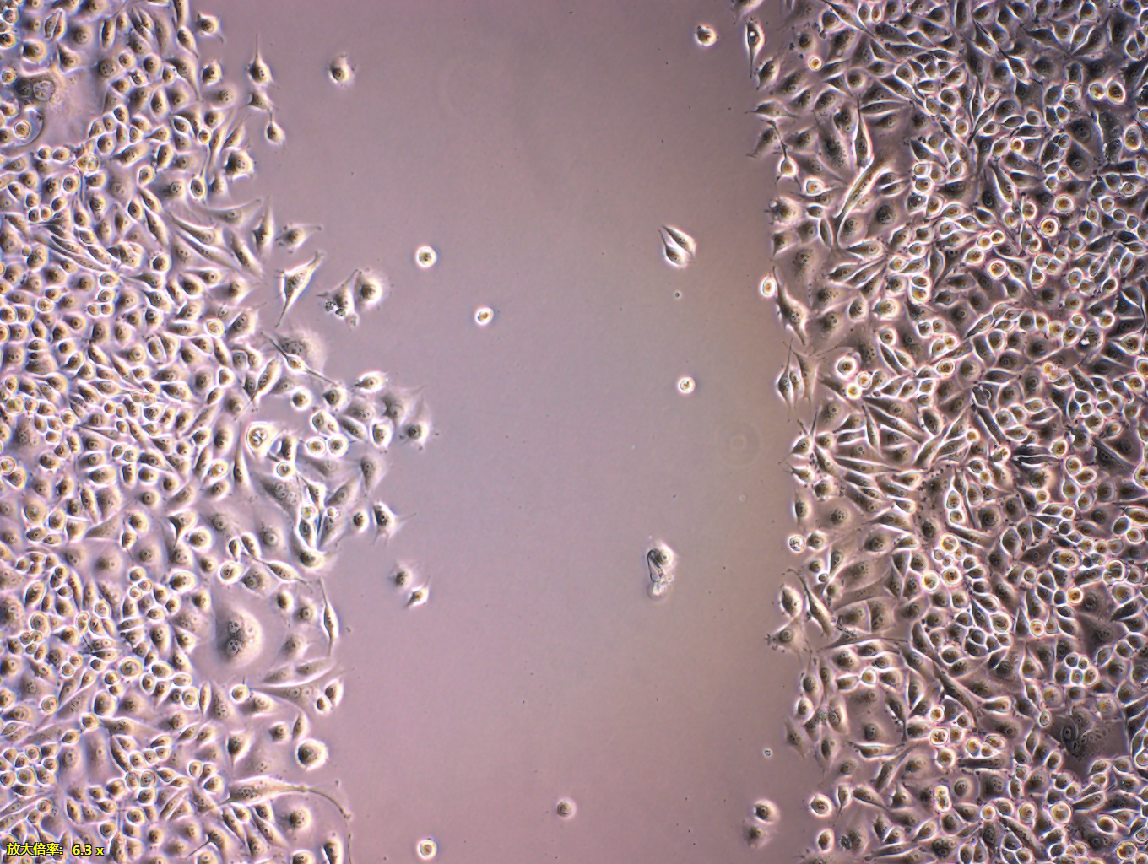

Supplement: Supplementary file 8 [file DataSheet5.ZIP › 24h/0-1.tif]

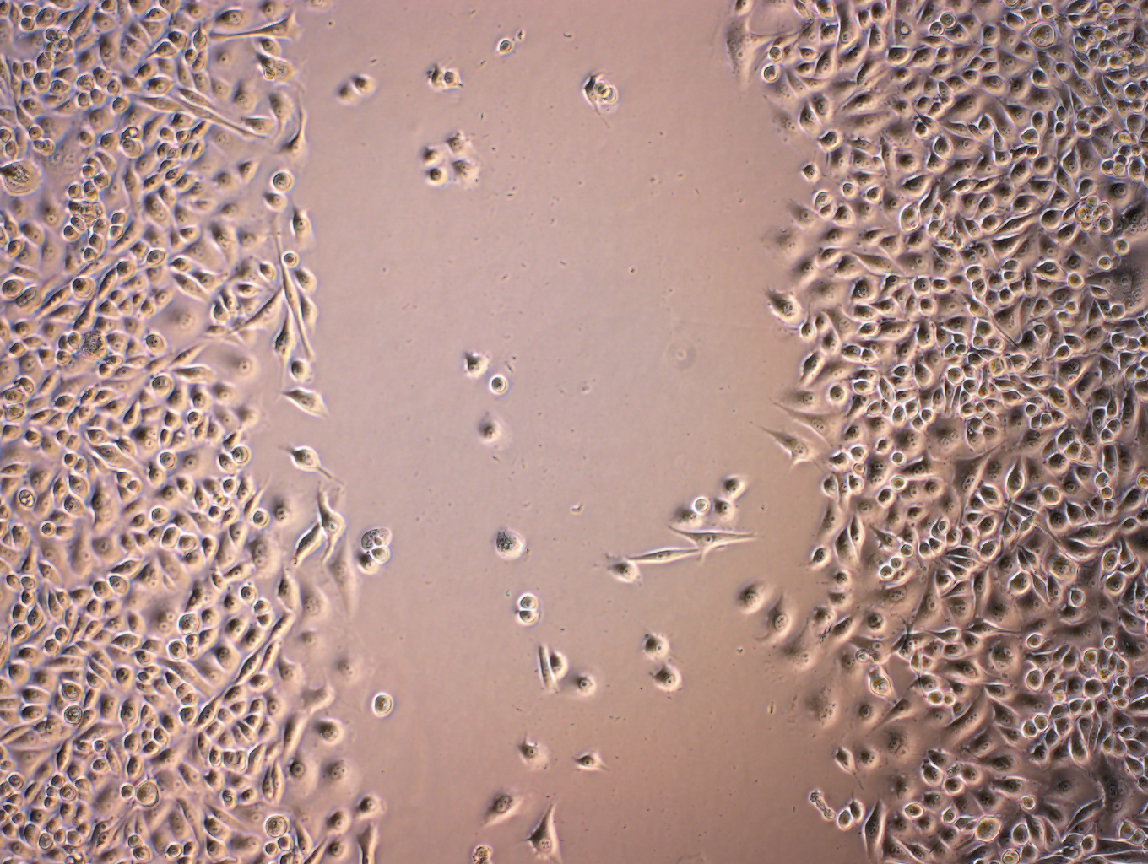

Supplement: Supplementary file 8 [file DataSheet5.ZIP › 24h/0-2.tif]

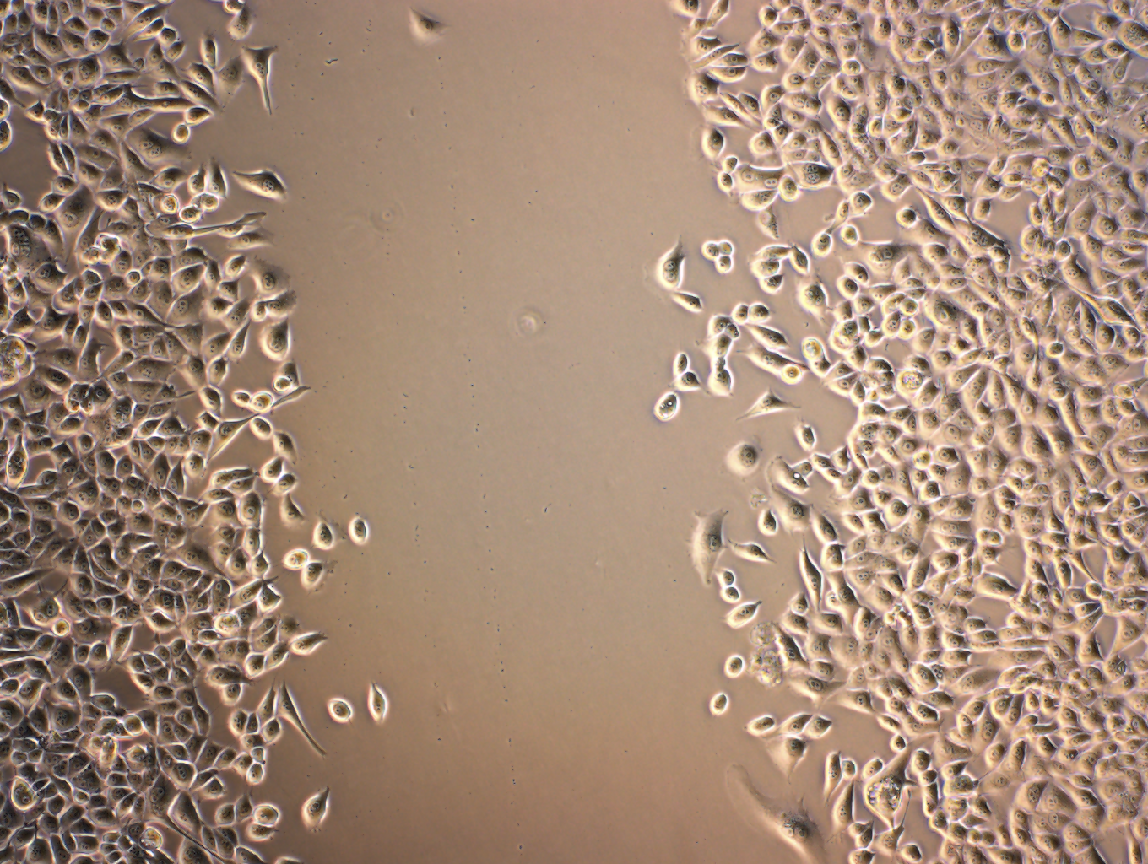

Supplement: Supplementary file 8 [file DataSheet5.ZIP › 24h/0.1-1.tif]

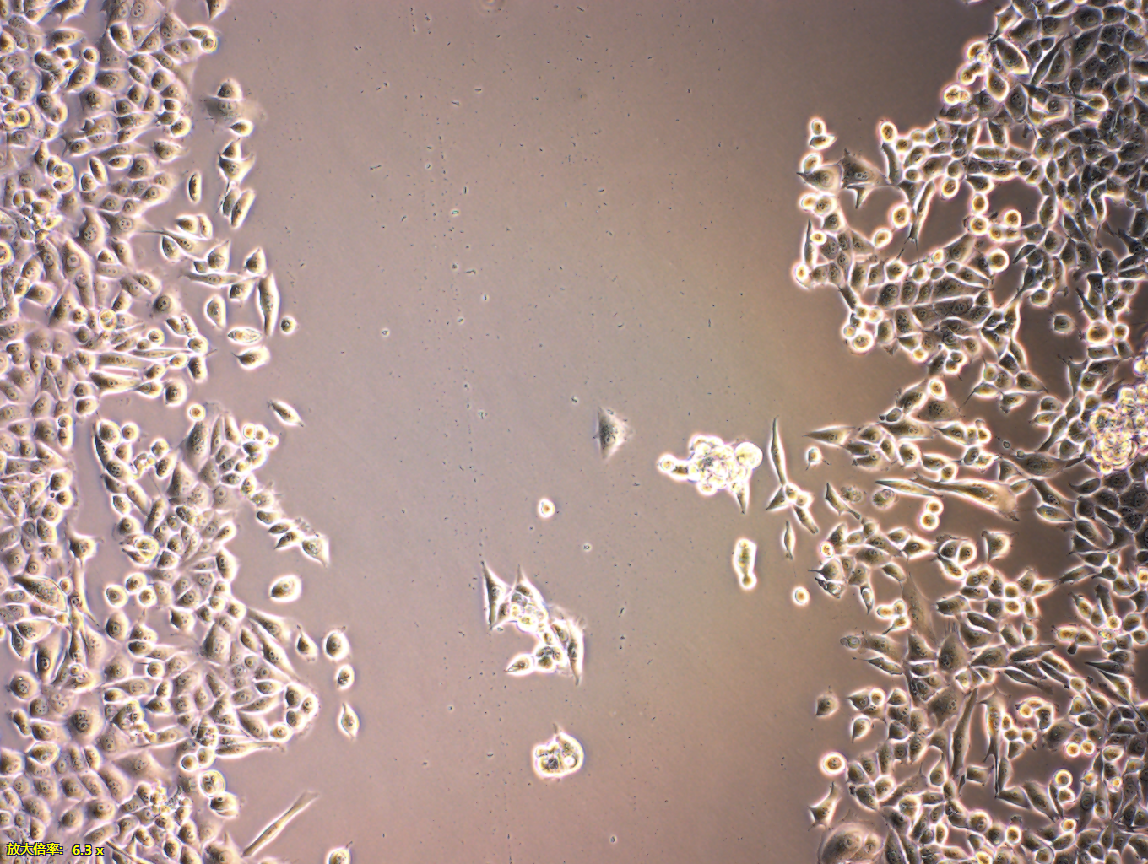

Supplement: Supplementary file 8 [file DataSheet5.ZIP › 24h/0.1-2.tif]
